# Analysis of host and viral nascent and steady-state RNA levels in a human neuronal model of herpes simplex virus 1 infection

**DOI:** 10.1371/journal.ppat.1014603

**Published:** 2026-09-16

**Authors:** Claire H. Birkenheuer, Terri G. Edwards, David C. Bloom, Joel D. Baines

**Affiliations:** 1 Department of Microbiology and Immunology, Cornell University Baker Institute for Animal Health, Ithaca, New York, United States of America; 2 Department of Molecular Genetics and Microbiology, University of Florida, Gainesville, Florida, United States of America; University of California, Berkeley, UNITED STATES OF AMERICA

## Abstract

We used precision nuclear run-on with sequencing (PRO-seq) in conjunction with quantitative RT-qPCR to map transcription, and transcript abundance in terminally differentiated LUHMES neurons before (mock) and after infection with Herpes Simplex Virus 1 (HSV-1). Initial PRO-seq analysis of cellular transcription demonstrated that the mock-infected differentiated LUHMES neurons expressed genes associated with differentiated neurons. This gene set included transcription of genes related to dopaminergic signaling, cell adhesion, and neuronal cell differentiation. By contrast, epithelial cells transcribed genes involved in cell cycle progression and DNA damage repair. PRO-Seq analysis of HSV-1-infected LUHMES neurons indicated only modest transcription of viral genomes. Additionally, genes of LUHMES neurons maintained RNA polymerase II (Pol II) promoter proximal pausing whereas we previously showed HSV-1 infection decreases promoter-proximal pausing on genes of epithelial cells. PRO-Seq and RT-qPCRanalysis were performed during a time course of infection, spanning both acute and latent phases. The results from the acute infection demonstrated slower rates of transcription, viral mRNA accumulation, and viral genome replication compared to undifferentiated, non-neuronal cells. RT-qPCR indicated that steady-state transcripts from all viral gene kinetic classes increased in abundance from 1 h to 24 h post-infection. This accumulation correlated with an increase in transcription on the same genes as demonstrated by PRO-seq. In contrast, the latent phase of infection was distinguished by high levels of transcription on all viral genes, but very low viral transcript abundance. These results indicate that viral gene transcription initiation does not correlate with viral transcript abundance during the latent phase, and suggest that blocks to RNA polymerase processivity and other components of the RNA life cycle likely contribute to maintenance of latency in LUHMES neurons.

## Author summary

Viral latency is a hallmark of all herpesvirus infections and arguably key to its high prevalence in human and animal populations. Previous studies showed minimal transcription of the Herpes Simplex Virus 1 genome during latency except for the latency associated transcript or LAT. Here we used a novel technique to identify transcriptional activity in a LUHMES neuronal culture model of HSV-1 latency. We found that HSV-1 transcription and viral genome replication were more restricted in LUHMES compared with those activities in undifferentiated infected epithelial cells. Surprisingly, the latent viral genome was more transcriptionally active than previously appreciated. While stable viral transcripts were in lower abundance in latency than in acute infection, transcriptional activity was higher. Unlike other viral transcripts, the LAT accumulated to high abundance during latency, despite only modest increases in transcription. These data suggest that HSV-1 latency is regulated through events following transcriptional initiation on many viral genes to limit production of viral mRNA.

## Introduction

Herpes simplex virus 1 infects and rapidly produces infectious virions in undifferentiated epithelial and fibroblast cells. Subsequent infection of enervating neurons leads to establishment of a lifelong latent infection of peripheral ganglia [1,2]. In undifferentiated epithelial cells and fibroblasts, HSV-1 gene expression occurs in a well-characterized temporal lytic cascade [3–10]. Each infected epithelial or fibroblast cell can yield hundreds of infectious particles ultimately resulting in cell death at the conclusion of the lytic cycle. Monolayers of cultured primary human skin fibroblasts as well as multiple epithelial cell lines have been routinely utilized to model productive HSV-1 infections [11–13].

As latency becomes established within neurons, the viral genomes circularize and are maintained as nuclear episomes [14–20]. During periods of latency, infectious virus remains undetectable until various cell signaling events lead to the induction of viral replication and full reactivation [21]. While animals infected with HSV-1 have been used to model latent infections, cultured neurons from multiple species, including humans, have been used more recently [16,20,22–24]. While studies utilizing animal models provide valuable insights into HSV-1 transcription regulation, the limited number of infected neurons in these systems constrains comprehensive transcriptional analyses with current technology.

Since the discovery of the nucleosome and the four core histones [25–27], our understanding of transcription has advanced dramatically. Steady-state mRNA levels in any given cell are the major driver of gene expression [28–34]. However, their steady-state levels reflect many mechanisms that regulate both transcription and RNA stability. These mechanisms include initiation by RNA polymerase II (Pol II), promoter-proximal pausing, release to elongation, processivity, termination, mRNA processing, nuclear export, and translational stability. Moreover, RNA degradation can occur following any of the transcriptional regulatory events [28,35,36].

Cellular and viral-encoded exonucleases degrade nascent RNA and mRNA to control gene expression. For instance, if not elongated after exiting the RNA polymerase complex, nascent RNA is rapidly degraded by the cellular exonuclease XRN2. Nascent RNA degradation can also occur at any location across a gene if the proper transcriptional-regulatory checkpoints have not been met [31,37–39]. The short half-life of nascent RNA mostly precludes accurate detection and identification using techniques like mRNA sequencing, total cell RT-qPCR, and northern blotting [40–42].

Precision nuclear run on with sequencing (PRO-seq) maps the production of nascent transcripts at a given time of interest. Additionally, it can be combined with techniques such as RT-qPCR and RNAseq to obtain valuable insights into mechanisms of RNA expression and regulation. The PRO-seq method incorporates high concentrations of biotinylated nucleotides used in the run-on reaction to terminate extension of nascent transcripts, marking the locations of actively transcribing Pol II. After extensive RNA fragmentation, Pol II activity is mapped to nucleotide resolution based on the 3’ end of the nascent transcript [43,44].

As a global technique, PRO-seq provides quantitative information on nascent RNA production from all genes, viral and cellular, and infers regulatory steps from initiation to termination [45]. This has an advantage over traditional Pol II ChIP-seq or cut-and-run experiments, which rely on antibody detection of the polymerase, suffer from high background pull-down of viral genomes, do not necessarily report active RNA polymerase II, and do not afford the resolution required to map promoter proximal pausing unless extensive exonuclease treatments are performed [46].

**Fig 1:**
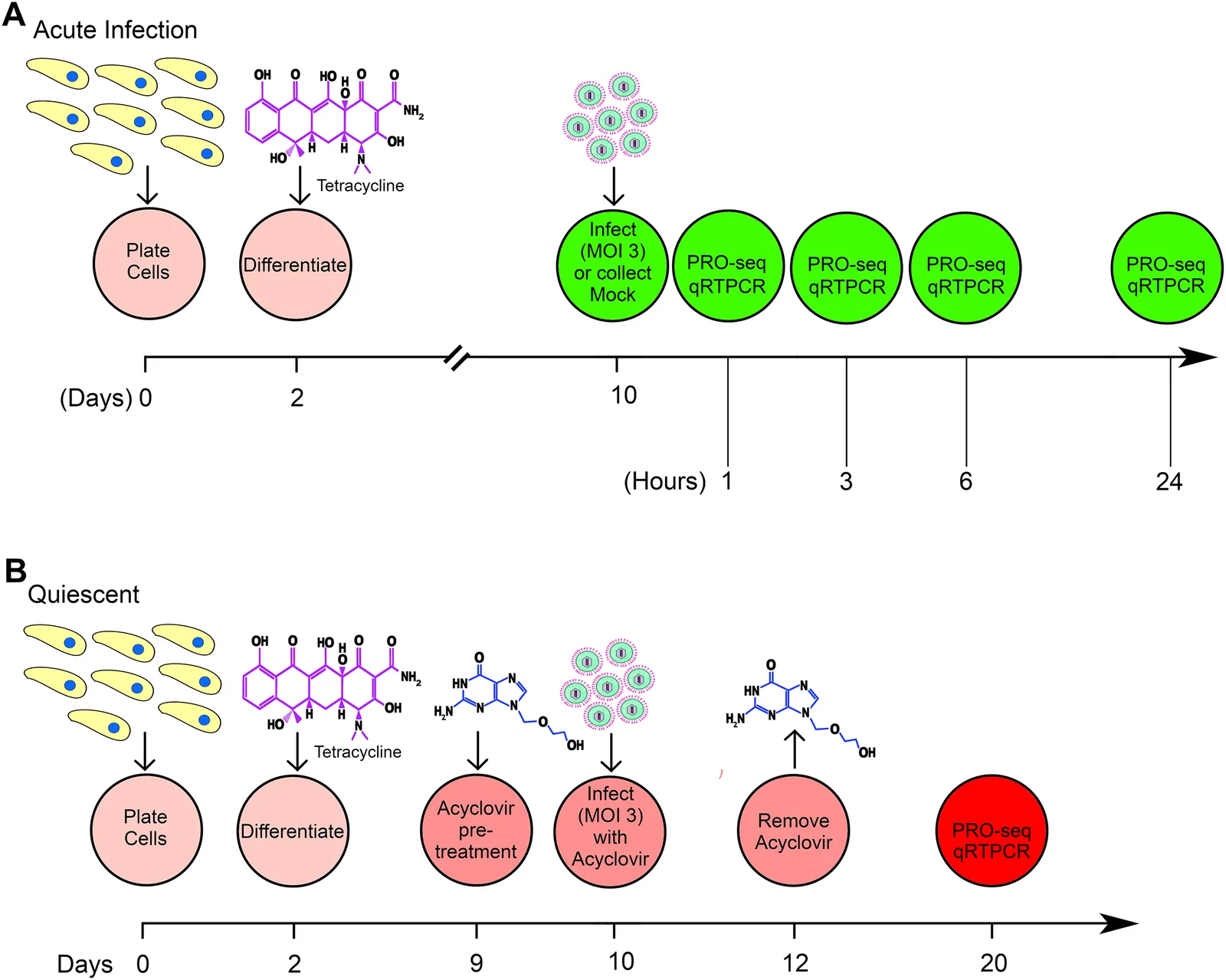
Time course of acute (A) and quiescent (B) infection in LUHMES. Mitotic LUHMEs were plated, and two days later the media was changed to include tetracycline required to induce differentiation into LUHMES. For both the acute and quiescent time courses, post-mitotic cells were infected at 8 days post differentiation (10 days post-plating) at an MOI of 3 with HSV-1 strain *17syn +* . However, cells for the quiescent experiments (B) were pre-treated with acyclovir starting at 7 days post-differentiation and continuing to 2 days post-infection or 10 days post-differentiation. At this time, acyclovir was removed, and the infection was allowed to progress for an additional 10 days. Mock-infected samples were collected at the time of infection, 10 days post-plating (8 days post-differentiation). Nuclei were isolated on ice and flash-frozen for PRO-seq and qRT-qPCR analysis at the indicated times. The acute time course had an n = 2 for each time point, and the quiescent samples had an n = 5.

We used PRO-seq to map the location of actively transcribing Pol II in differentiated LUHMES neurons. The LUHMES neurons were interrogated under uninfected (mock) conditions, and during a time course of HSV-1 infection that included an acute phase and a latent phase, which has been previously characterized as having very limited viral lytic activity (Fig 1) [22,47–51]. As a baseline and to demonstrate the background transcriptional differences between cell types that support full lytic HSV-1 infections, we compared the transcriptional profile of undifferentiated epithelial (HeP2) cells with LUHMES neurons under mock infection conditions. Unsurprisingly, uninfected HeP-2 cells and LUHMES neurons produced nascent RNA from different gene sets consistent with their known respective origins and phenotypes.

Interestingly, upon infection with HSV-1, LUHMES neurons were largely resistant to the HSV-1-induced transcriptional changes observed in previous studies using HeP-2 cells. For instance, we find that HSV-1 genome replication is restricted in LUHMES neurons compared with the extremely efficient replication during HeP-2 cell infections. This very slow, limited accumulation of viral genomes within LUHMES neurons likely affects the scope and rate of transcriptional activity in the two cell types.

Perhaps the most interesting insight from this study involves the unexpected transcriptional activity measured by Pro-seq on the viral genomes during HSV-1 latency in LUHMES neurons. Surprisingly, virtually every viral gene demonstrated this transcriptional activity with significantly higher levels than those observed following a 24-hour acute infection. Intriguingly, RNA polymerase activity on the viral Immediate Early genes, *α*4 and *α*0, along with some other genes mapping to the internal repeat region of the viral genome, was restricted upstream of the promoter-proximal pause sites with minimal transcriptional activity through the gene bodies. In contrast, transcription through these same gene bodies was readily apparent after a 24-hour acute infection and coincident with higher abundance of steady-state transcripts than what was measured during latency by RT-qPCR. Unsurprisingly, we found the most abundant steady-state transcripts during latency in LUHMES neurons were the latency-associated transcripts (LATs). Intriguingly and in stark contrast to the observed Pol II transcriptional regulation of the lytic genes during latency, the LAT region was decidedly less transcriptionally active. Due to the lariat structure and inherent stability of the LAT 1.5 and 2kb introns, accumulation of RNA from this region would logically involve additional regulatory mechanisms, such as high stability or splicing, aside from Pol II activity. Collectively, these data demonstrate large differences in the capacity for viral genome replication, transcriptional regulation, and timing of viral gene expression following HSV-1 infection in LUHMES neurons compared to epithelial cells. Importantly, the data also indicate that promoter-proximal pausing on critical viral regulatory genes is important for maintaining the viral latent state and/or poising the genomes for reactivation in LUHMES neurons.

## Results

### Quality control and normalization of PRO-seq data

We assessed nascent RNA production in LUHMES neurons at ten days post-differentiation using established protocols described in the methods [5,22,48,51,52]. All samples were independent biological replicates from initial infection until harvesting. Two cultures were analyzed for each acute time point, and five cultures analyzed from the latent time point. We first looked globally at the PRO-seq dataset to determine if the libraries were of high quality (Table 1). Anticipating low viral reads, we reduced spike-in amount compared to previous PRO-seq experiments [5,13,45,46,53,54]. However, the number of reads aligning to the hg38 genome was more consistent for all samples than were reads from the drosophila spike-in (Table 1). Drosophila spike-in reads varied between 308,233–4,199,174 whereas reads aligning to the hg38 genome ranged from 9,201,724–75,402,261. The 1hr acute rep 1 sample had the lowest number of hg38 reads, but also the lowest number of reads aligning to the viral genome. Thus, based on the data in Table 1, the lower spike in volume was deemed unnecessary and the data was normalized to the more consistent human reads per million to avoid the artificial introduction of variance.

**Table 1:** Comparison of reads aligning to each genome from each sample.

Sample	Number of Reads		
	*Homosapien* hg38	*Drosophila* dm3	HSV-1 17 Syn+
Mock rep1	16043277	308233	0
Mock rep 2	19338557	593400	0
1hr acute rep1	9201724	331929	936
1hr acute rep 2	14852452	654284	1670
3hr acute rep1	31633019	2240388	4340
3hr acute rep2	33902296	1326244	6290
6hr acute rep1	37065855	1073625	28854
6hr acute rep2	14868415	450657	10906
24hr acute rep1	14336567	454911	89710
24hr acute rep2	17077758	482487	103444
Latent exp1 rep1	55107310	4199174	2177792
Latent exp1 rep2	74134994	3443024	2127196
Latent exp1 rep3	45489296	3368729	1379522
Latent exp2 rep1	75402261	733739	1133039
Latent exp2 rep2	72436952	625741	946753

### Cell-type specific differences in nascent RNA production were identified for LUHMES neurons and Hep2 cells

**Fig 2:**
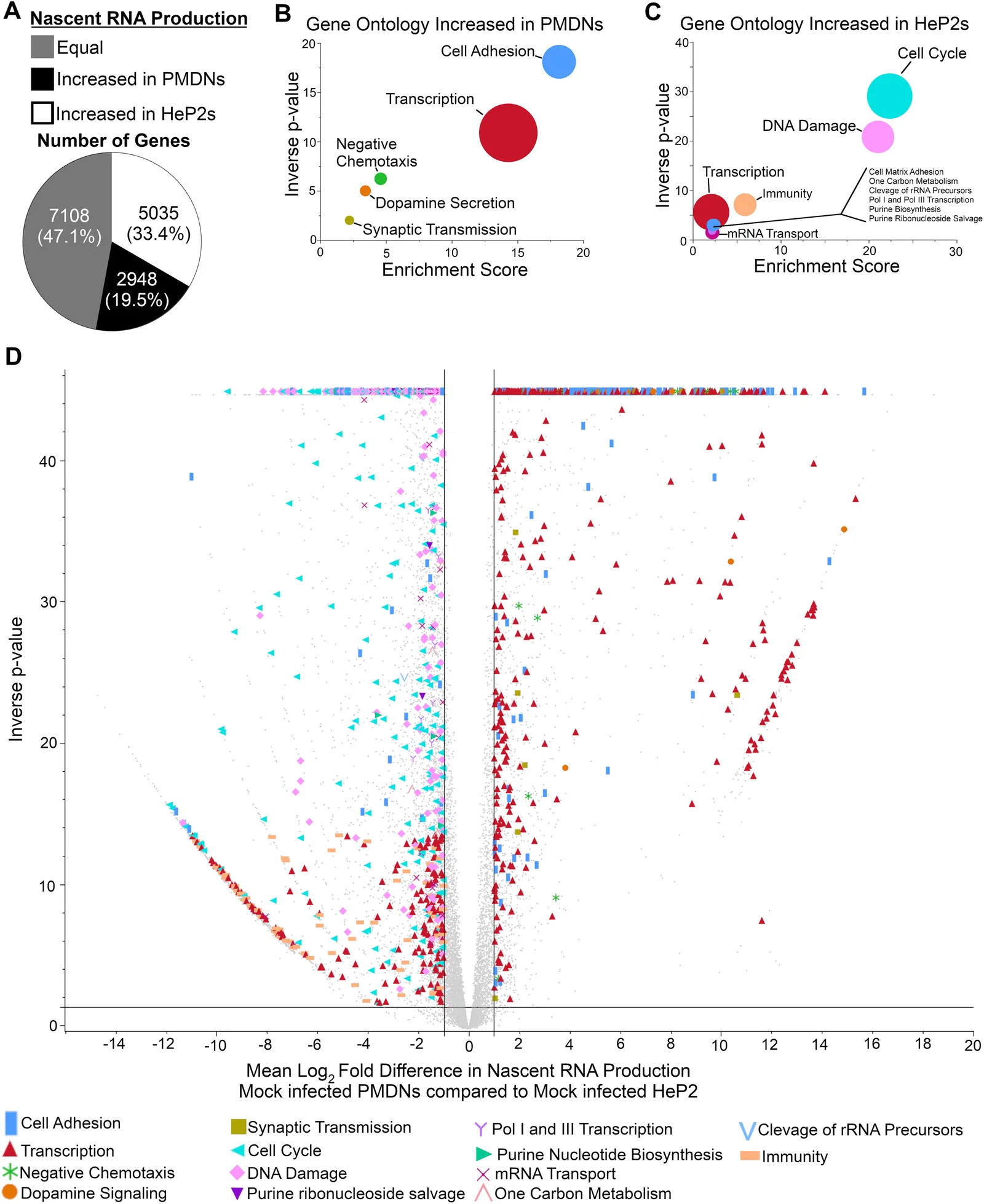
HSV-1 induced changes in cellular gene transcription. **(A)** A pie chart summary of the DESeq2 analysis comparing nascent RNA production from cellular genes in mock-infected LUHMES (experimental) or HeP-2 cells (control). The HeP-2 data sets were previously published and re-analyzed for this experiment [46]. Genes with increased transcription in LUHMES are in black, genes with increased transcription in HeP-2 cells are depicted in white, and genes with equal transcription in both cell types are in gray. Percentages refer to the number of genes within each category out of the number of analyzable genes. **(B and C)** Bubble plots depicting the results from a Database for Annotation, Visualization, and Integrated Discovery (DAVID) functional annotation analysis [99,102] of genes with increased nascent RNA production in LUHMES compared to HeP-2 cells. Categories were considered significant if they had an enrichment score ≥ 0.5 and a p-value < 0.05. The bubble size represents the number of genes belonging to each category. The Gene Ontology Biological Process and UniProt Key Words Biological Process databases were used for the functional annotation. **(D)** Volcano plot of the DESeq2 comparison. Genes belonging to the different categories identified by the DAVID ontology analysis are depicted in color, as indicated in the legend at the bottom of the Fig. Genes not grouped in the DAVID analysis are shown as gray dots.

A DESeq2 analysis was performed comparing read densities obtained from whole gene probes between mock-infected LUHMES neurons and previously published mock-infected HeP2 cells [46]. As shown in Fig 2A, almost half of the analyzable genes (defined as possessing >200 reads per gene and >0.01 read density) had Pol II activity in both cell types, whereas up to a third and 20% of analyzable genes had increased Pol II activity in HeP-2 cells and LUHMES neurons, respectively.

Importantly, transcription in LUHMES neurons was associated with genes conferring markers of differentiated dopaminergic neurons, synaptic transmission, dopamine secretion, negative chemotaxis of axon growth, cell adhesion and inhibition of the cell cycle, consistent with the previously characterized phenotype of these cells (Fig 2B). Additionally, more than 390 Pol II-specific transcription factor genes were transcribed exclusively in LUHMES neurons (Fig 2B and 2D) compared to HeP2 cells (Fig 2C and 2D). Interestingly, many transcription factors necessary for efficient HSV-1 replication in epithelial cells (e.g., *HCF1*, *NF*κ*B*, *c-FOS*, *EGR1*, *KLF15*, and *JUN* were not transcribed as robustly in LUHMES neurons (Table 2) [55–62].

**Table 2:** Comparison of nascent RNA production on cellular genes involved in HSV-1 replication and/or cellular Pol II transcription in LUHMES compared to Hep2 cells under mock infection conditions.

Cellular Factors/ Complexes	Subunit(s)-Encoding Gene(s)	Nascent RNA production in LUHMES compared to HeP-2s	References
**ND10s**	PML	Same	
	**SP100**	**Down**	
	**IFI16**	**Down**	
	ATRX	Same	
	DAXX	Same	
**TFIID**	TAF1	Same	
	TAF2	Same	
	TAF3	Same	
	TAF4	Same	
	**TAF5**	**Down**	
	TAF6	Same	
	TAF7	Same	
	**TAF8**	**Down**	[103]
	**TAF9**	**Down**	[103,104]
	TAF9B	Same	
	TAF10	Same	
	TAF11	Same	
	TAF12	Same	
	TAF13	Same	
	TBP	Same	
TFIIA	GTF2A1	Same	
	GTF2A2	Same	
TFIIB	GTF2B	Same	
TFIIF	GTF2F1	Same	
	GTF2F2	Same	
TFIIE	GTF2E1	Same	
	GTF2E2	Same	
**TFIIH**	ERCC3	Same	
	ERCC2	Same	
	**GTF2H1**	**Down**	[104–106]
	GTF2H3	Same	
	GTF2H5	Same	
	CDK7	Same	
	CCNH	Same	
	MNAT1	Same	
**Relevant Sequence-Specific Transcription Factors**	**TP53**	**Down**	
	**Myc**	**Down**	[46]
	SP1	Same	
	SP3	Same	
	SLC22A1	Same	[107,108]
	**HCFC1**	**Down**	[55,56,109–111]
	**MYBBP1A**	**Down**	[112–115]
	RELA	Same	[57,58,116]
	**REL**	**Down**	[57,58,116]
	**RELB**	**Down**	[57,58,116]
	**NFKB1**	**Down**	[57,58,116]
	**NFKB2**	**Down**	[57,58,116]
	**IRF3**	**Down**	[117–119]
	**EGR1**	**Down**	[46,120]
	**KLF15**	**Down**	[62,121]
	**FOS**	**Down**	[46,122,123]
	FOSB	Same	
	**FOSL1**	**Down**	[46]
	**FOSL2**	**Down**	[46]
	JUN	Same	
**Mediator**	**MED13L**	**Up**	[104,124,125]
	**MED24**	**Up**	[104,112,126]
	**MED10**	**Down**	[104,127]
	**CDK8**	**Up**	[104,128–131]
	CCNC	Same	
	MED1	Same	
	MED4	Same	
	MED6	Same	
	MED7	Same	
	MED8	Same	
	MED9	Same	
	MED11	Same	
	MED12	Same	
	MED13	Same	
	MED14	Same	
	MED16	Same	
	MED17	Same	
	MED18	Same	
	MED19	Same	
	**MED20**	**Down**	[104,132,133]
	MED21	Same	
	MED22	Same	
	MED23	Same	
	MED25	Same	
	**MED26**	**Down**	[134,135]
	MED27	Same	
	MED28	Same	
	MED29	Same	
	**MED30**	**Down**	[104,136–138]
	MED31	Same	
**SWI/SNF**	**ARID1A**	**Up**	[87,104]
	**ARID1B**	**Up**	
**Pol II**	POLR2A	Same	
	POLR2B	Same	
	**POLR2C**	**Down**	[5,104,139–141]
	**POLR2D**	**Down**	
	POLR2E	Same	
	POLR2F	Same	
	POLR2G	Same	
	POLR2H	Same	
	**POLR2L**	**Down**	
	**POLR2J**	**Down**	
	**POLR2K**	**Down**	
**PAF1C**	PAF1	Same	
	**CTR9**	**Down**	[66,142,143]
	**CDC73**	**Down**	
	RTF1	Same	
	LEO1	Same	
**DSIF**	**SUPT4H1**	**Down**	[144–146]
	SUPT5H	Same	
**NELF**	**NELFA**	**Up**	[91,144,147]
	NELFB	Same	
	**NELFCD**	**Down**	
**7SKsnRNP**	CDK9	Same	
	CCNT1	Same	
	CCNT2	Same	
	**HEXIM1**	**Down**	[92,148]
	LARP7	Same	
	**MEPCE**	**Down**	
BRD4	BRD4	Same	
**FACT**	SSRP1	Same	
	**SUPT16H**	**Down**	[53,104,144,149,150]
IWS1	IWS1	Same	
SPT6	SUPT6H	Same	
**Integrator**	**INTS10**	**Down**	[104,151]
	INTS5	Same	
	INTS8	Same	
	INTS6	Same	
	PPP2R1A	Same	
	PPP2R1B	Same	
	PPP2CA	Same	
	**INTS4**	**Down**	
	INTS9	Same	
	**INTS7**	**Down**	
	INTS1	Same	
	INTS2	Same	
	INIP	Same	
	**NABP1**	**Down**	
	NABP2	Same	
**Co-transcriptional**	SRPK1	Same	
**Splicing and mRNA export**	**ALYREF**	**Down**	[152–155]
	THOC1	Same	
	**THOC2**	**Down**	
	**THOC5**	**Up**	
	**THOC6**	**Down**	
	THOC7	Same	
	CHTOP	Same	
	SRRT	Same	
	NCBP1	Same	
	NCBP2	Same	
	**EIF4A3**	**Down**	
	MAGOH	Same	
	RBM8A	Same	
	NXF1	Same	
	**NXT1**	**Down**	
**Cleavage, Polyadenylation, and Termination Machinery**	CPSF1	Same	
	CPSF2	Same	
	**CPSF3**	**Down**	[12,45,155]
	**CPSF4**	**Down**	
	WDR33	Same	
	**CSTF1**	**Down**	
	**CSTF2**	**Down**	
	CSTF2T	Same	
	CSTF3	Same	
	SYMPK	Same	
	**CLP1**	**Up**	
	PCF11	Same	
	NUDT21	Same	
	CPSF6	Same	
	CPSF7	Same	
	**PABPN1**	**Up**	
	PAPOLA	Same	
	PAPOLG	Same	
	XRN2	Same	
	SETX	Same	

HeP-2 cells, on the other hand, displayed increased transcription of genes associated with cell cycle progression, DNA damage, and innate immunity. Other comparatively active categories in the HeP-2 cells included carbon metabolism (specifically folic acid synthesis), cleavage of rRNA precursors, Pol I and Pol III transcription, mRNA transport, and purine biosynthesis and salvage (Fig 2C and 2D).

Collectively, these data demonstrate that LUHMES neurons display a transcriptional profile expected of post-mitotic neurons following terminal differentiation, including the induction of dopaminergic signaling and little to no transcription of genes associated with immunity or cell cycle progression. Furthermore, these gene pathways varied substantially from the transcriptionally active genes identified in HeP-2 cells. Thus, as expected, and similar to human infections, HSV-1 encounters an entirely different cellular and transcriptional environment upon entry into these two human cell types (Fig 2D).

### Differences in nascent RNA production on genes involved in HSV-1 replication and/or Pol II transcription

Within the DESeq2 analysis, some genes were mapped that did not cluster with a known biological function in the ontology tool DAVID (Fig 2D, gray dots). Previous studies identified several host cell factors that enhance or inhibit HSV-1 infections. These cellular factors are known to regulate transcription and/or chromatinization of incoming viral genomes. To facilitate the analysis, we compiled a list from published reports of genes whose products are part of Pol II transcriptional complexes and known to interact with HSV-1 proteins, or to comprise critical components of the cellular Pol II transcriptional machinery upon which HSV-1 transcription depends. We then noted whether nascent RNA production from these genes was increased or decreased in our two cell models (Table 2).

Of the 169 genes comprising the HSV-1 and Pol II-specific gene subsets, 111 had similar nascent RNA production between mock infected LUHMES neurons and HeP-2 cells, 49 had decreased nascent RNA production in LUHMES neurons, and 9 had increased nascent RNA production in HeP-2 cells (Table 2). The table also describes the roles of the protein of interest during HSV-1 infection and/or Pol II transcription, any known interactions with HSV-1 encoded proteins, and whether they are known to regulate HSV-1 infections. Examples include genes encoding components of ND10 nuclear bodies, TFIID, TFIIH, Mediator, Pol II, PAF1C, DSIF, NELF, the 7SKsnRNP, FACT, Integrator, and components of complexes mediating RNA processing, including mRNA splicing and export, RNA cleavage, and polyadenylation (Table 2). Collectively, these data suggest that LUHMES neurons have lower Pol II activity on many genes that encode proteins known to enhance HSV-1 infections (Fig 2, and Table 2). These variations likely contribute to differences in viral transcription, replication, and infection between the two cell types.

### HSV-1 alteration of neuronal transcription during acute and latent infection of LUHMES neurons

During the course of an HSV-1 infection, chromatin immunoprecipitation, PRO-seq analysis, and phase-separation experiments clearly demonstrate repurposing of a large proportion of cellular Pol II to viral genomes by 6 hours post-infection in mitotic cells with either an epithelial or fibroblast expression profile [46,63–65]. This repurposing is estimated to approach a complete loss from cellular genes, as estimated by ChIP-seq, or as much as a 70% loss as estimated by PRO-seq experiments [5,46,64]. As demonstrated by the number of human PRO-seq reads shown in Table 1, repurposing of Pol II to viral genes at these levels does not occur in LUHMES neurons neither during the acute times post-infection, nor at the latent time point.

**Fig 3:**
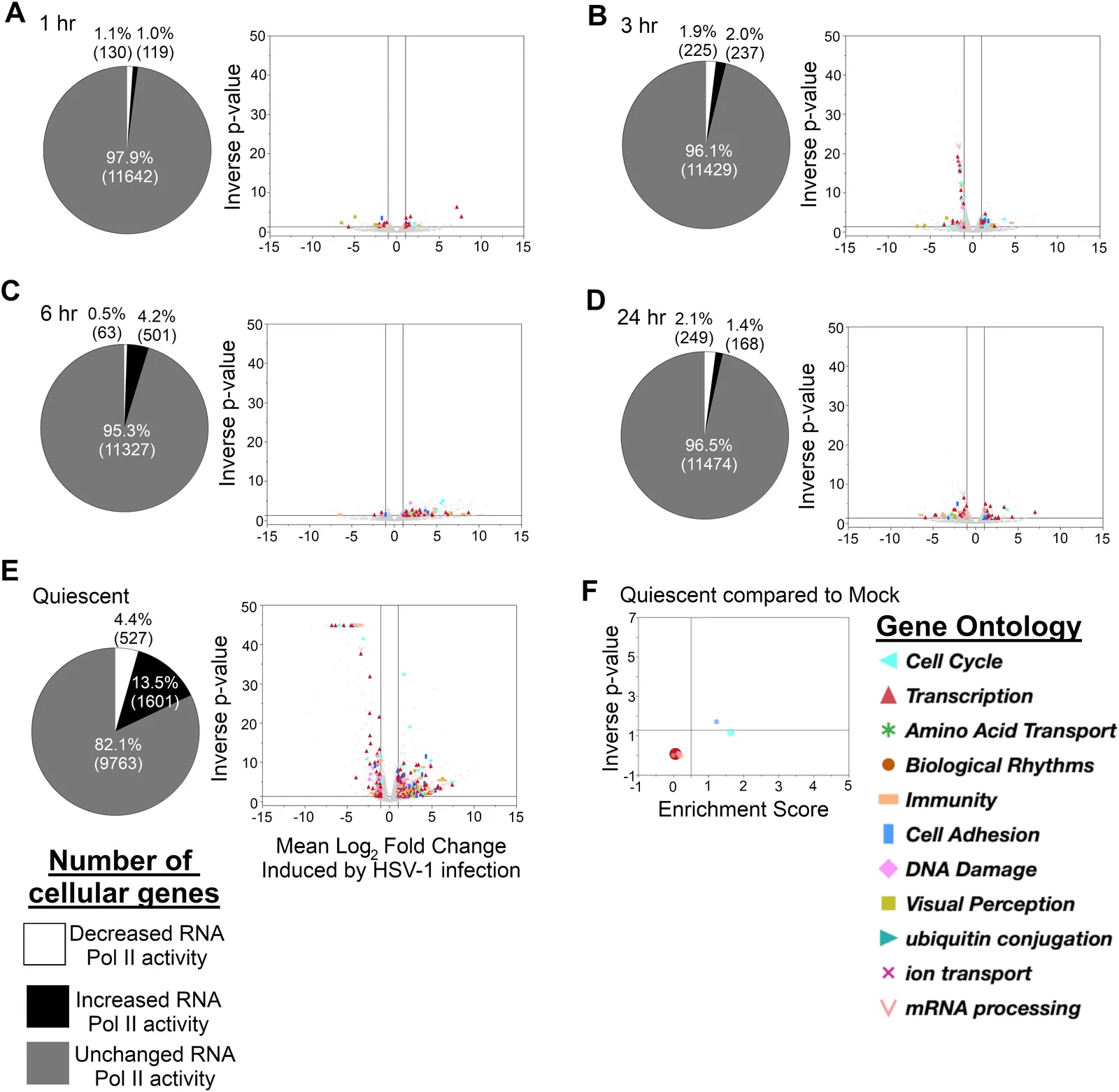
Pie charts and volcano plots demonstrating HSV-1 alteration of Pol II activity on cellular genes over time in LUHMES. A DESeq2 analysis was used to identify LUHMES genes with significantly increased or decreased Pol II activity compared to the corresponding LUHMES mock infection (p-value ≤0.05 and an HSV-1 induced mean log2 fold change of ≤ -1 or ≥ 1). Pie charts in each panel indicate the number of genes regulated similarly or differently at each time point. In the corresponding volcano plots, differentially regulated genes are designated by symbols indicating their DAVID gene ontology association (color coded in the key at lower right). Data was generated from samples taken after the following times post-infection: **A**, 1 hour; **B**, 3 hours; **C**, 6 hours; **D**, 24 hours; **E**, Quiescent infection. **F.** The same gene ontology labels are used to compare entire differentially regulated categories in mock infected LUHMES compared to quiescently infected LUHMES cells.

To better quantify this effect and to compare it with previously published analyses, we performed a DESeq2 analysis comparing the reads from whole gene probes between the mock infected LUHMES neuronal cultures and the infected cultures (Fig 3). Genes were considered to have either increased or decreased nascent RNA production if there was a Log_2_ fold change of either -1 or 1 with an accompanying p-value from the DESeq2 analysis of 0.05 or less. For each time point post-infection (Fig 3A-3E) we calculated the number of genes with increased, decreased or no change in overall nascent RNA production. Differentially expressed genes were then analyzed for common biological functions using DAVID gene ontology, which is shown in the accompanying volcano plot for each time.

We found that less than 5% of neuronal genes were altered at any given time during the acute phase of infection, with around 11,000 neuronal genes consistently exhibiting no observable difference in nascent RNA production when compared to the mock infected samples (Fig 3A-3D). The latent time point showed the most striking neuronal gene transcriptional dysregulation, with 2,128 (17.9%) of 11891 neuronal genes altered when compared with the mock infections (Fig 3E). This represents significantly fewer altered genes than previously observed following HSV-1 infections of epithelial and fibroblast cells in which nascent transcription of up to 70% of cellular genes is altered by 6 hours post infection [5,46].

When the small proportion of LUHMES neuronal genes with transcriptional dysregulation induced by HSV-1 infection were subjected to DAVID gene ontology analysis, it was apparent that they belonged to categories that were more transcriptionally active in mock infected HeP2 cells than in LUHMES neurons. This was particularly evident during latency (Fig 3E), where we observed modest increases in transcriptional activity on genes involved in cell cycle regulation, certain transcription factors, immunity, and DNA damage response due to HSV-1 infection. However, only the cell adhesion category was significantly altered based on enrichment score and p-value (Fig 3F). Together, these data indicate that LUHMES neuronal genes are either inherently resistant to HSV-1 induced changes to the transcriptional machinery, and/or that reduced expression of viral transcriptional regulators largely preclude these changes.

### HSV-1 infection increases promoter-proximal pausing on LUHMES neuronal genes

HSV-1 infections of epithelial cells cause decreases in promoter proximal pausing on cellular genes, which is associated with the massive HSV-1 induced perturbation of transcription in these cells [11,12,46]. Considering that LUHMES neurons were found to be largely resistant to these same transcriptional defects, we asked whether HSV-1 induced alterations of promoter proximal pausing also occurred in these cells.

**Fig 4:**
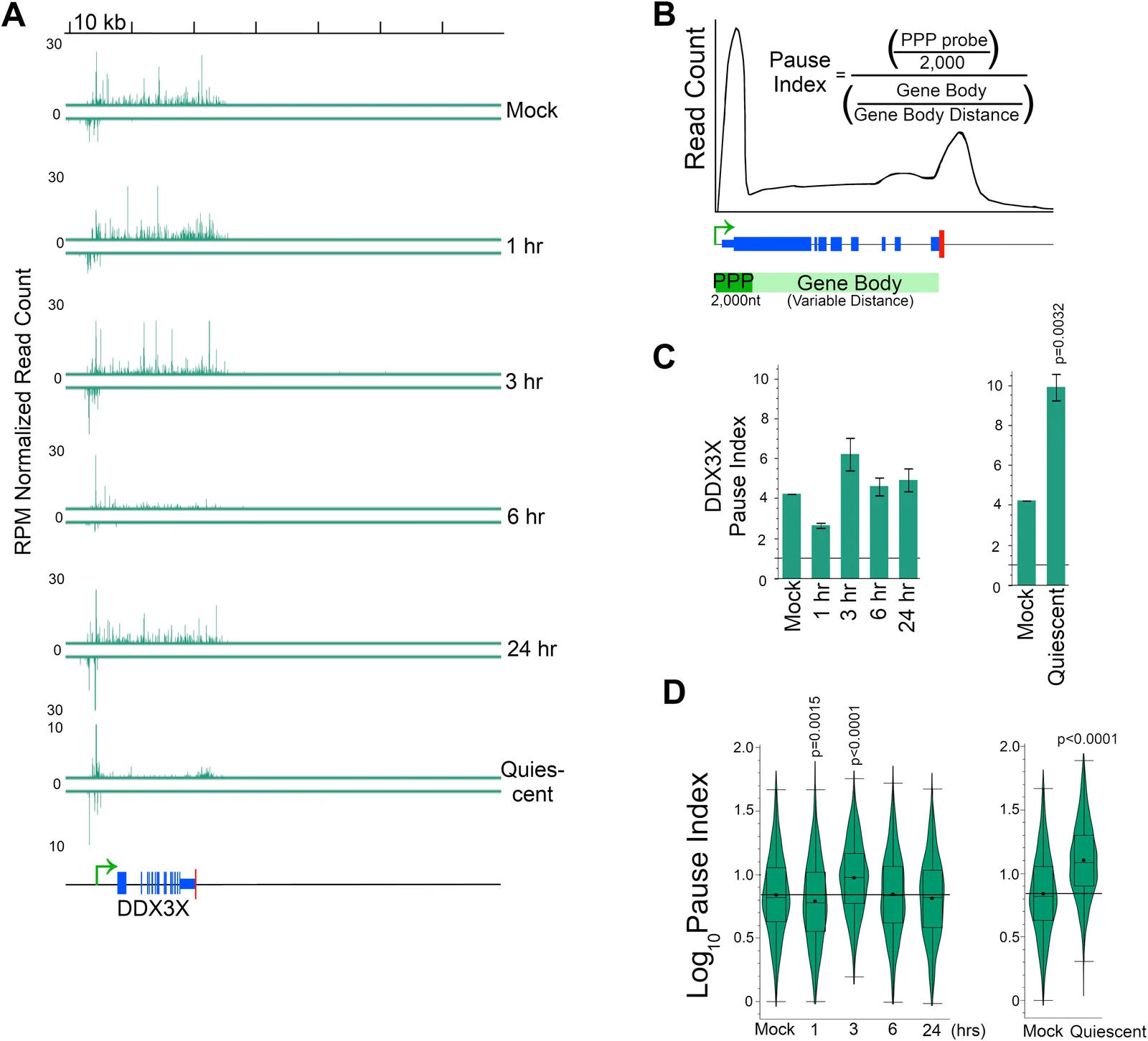
**(A)** IGV views of nascent RNA production from the *DDX3X* locus in LUHMES at different time points post HSV-1 infection. The mean of the total human RPM normalized read counts are shown for each bin across the 70kb *DDX3X* locus, with the data from the replicates overlaid in the viewer. The data from the *DDX3X* coding strand (top strand) and the antisense strand (bottom strand) is displayed for each time point. The settings were adjusted so each bin’s maximum/minimum value was presented, and the y-axes were adjusted to accommodate peak heights. **(B)** A description of the probes and formulas for the cellular gene pause index calculation are shown. **(C)** The pause index calculations for *DDX3X* at each time point post infection are shown. The data presented are the mean pause indices for each time point, with the error bars showing the standard error of the mean from each biological replicate. Statistical analysis was performed using a one-way ANOVA followed by a Tukey post hoc analysis. **(D)** A meta-analysis of the pause index of LUHMES genes over the HSV-1 infection time course. A total of 579 genes were analyzed, as these genes were transcribed in both HeP-2 cells and LUHMES in mock conditions, where they held the typical pattern of Pol II distribution--a high 5’ index and a processivity index less than 1 [45].

As a first step we examined transcription of the cellular gene, *DDX3X* (Fig 4). This gene was chosen because its product has been shown to be involved in efficient infection of epithelial cells and fibroblasts, it is robustly transcribed in LUHMES neurons, and exhibits promoter-proximal pausing. We first viewed the RPM-normalized reads aligning to *DDX3X* over the LUHMES neuron time course using the IGV viewer, adjusting the y-axis for optimal visualization for each sample set (Fig 4A). The 5’ promoter-proximal pause peak in IGV was most pronounced during latency, demonstrating that HSV-1 infection increases promoter-proximal pausing on this gene.

To quantify this effect, we used a pause index with a 5’ PPP window of 2000 nt, as this window most closely matched the base-width observed in IGV (Fig 4B). This was performed for *DDX3X* for each time point and plotted (Fig 4C). We quantified slight, but non-significant alterations in promoter-proximal pausing on *DDX3X* during the acute phase of infection, but a significant increase over mock infection in promoter proximal pausing during latency (Fig 4C).

Next, we used a meta-analysis to measure promoter-proximal pausing on mRNA-coding genes that were similar in length to *DDX3X*, using the same formula used for analysis of *DDX3X* (Fig 4D). The genes analyzed can be found in S1 Table. and were included if they all had measurable promoter proximal pausing in HEp-2 cells, and were annotated as being single, non-overlapping gene loci lacking a transcriptionally active neighboring gene within 20 kb downstream of their annotated polyA site. This analysis was done to accommodate differences in the heights of different promoter-proximal pause peaks on genes of varying lengths [66]. Including more genes in the meta-analysis rendered the small variations observed on the *DDX3X* at the 1hr and 3hr acute infection time points statistically significant, with a decrease in cellular gene promoter-proximal pausing at 1hr post infection, followed by a significant increase over mock at 3hr. For the remainder of the acute time course, the meta-analysis showed that the cellular genes had similar levels of promoter proximal pausing as observed for the mock infection. However, the latency time point showed that many genes had increased promoter proximal pausing compared to the mock infection, similar to *DDX3X*. Thus, transcription from LUHMES neuronal genes was altered by a prolonged HSV-1 infection (Fig 4D). It is interesting to note that this increase in pausing on LUHMES neuronal gene promoters differs substantially from the HSV-1 induced decreases in pausing observed on most epithelial and fibroblast genes (S1 Fig) [11,46,53,67].

### Viral DNA replication is restricted in LUHMES neurons

**Fig 5:**
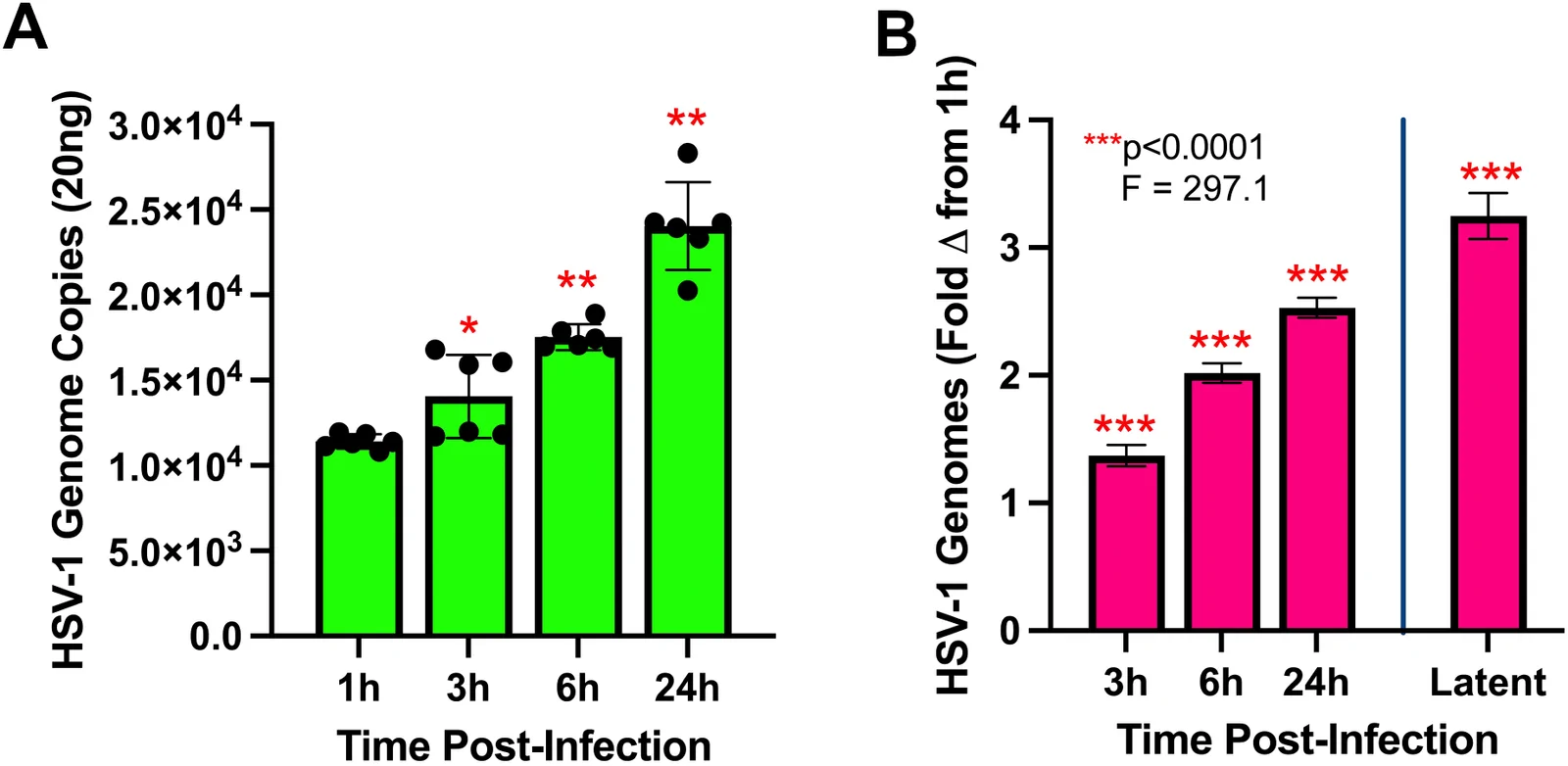
A qPCR analysis of steady-state viral genome copy number in LUHMEs derived neurons. Total DNA was extracted following the same time course of infection and relative HSV-1 genome copies were calculated by qPCR from 20ng input. **(A)** A small but significant increase in viral genome copy number was measured at 3hpi (1.232-fold change from 1hpi; *p = 0.0448) and viral genome replication proceeded with extremely slow kinetics from 6hpi (1.537-fold change from 1hpi; **p=<0.0001) and 24hpi (2.108-fold change from 1hpi; **p=<0.0001). Statistical analyses: Unpaired t test with Welch’s correction for each time point compared to 1h; n = 2 independent experiments with 3 biological replicates. **(B)** We performed the same time course of acute infection in the presence of acyclovir and found modest increases in viral genomes like that seen from infections of LUHMES cultures in the absence of acyclovir. At the latency timepoint (10d; 48h initial acyclovir pulse) viral genomes were increased 3-fold from the 1h acute timepoint, indicating acyclovir treatment of LUHMES cultures does not block viral replication during the acute phase of infection. The fold change in steady-state viral RNA levels from 24hr to Latency can be found in Table 4. The error bars represent the standard deviation from two (3h-24h) to three (latent) independent experiments with three biological replicates each.

To assess viral DNA replication, we plotted the mean level of viral DNA using RT-qPCR analysis of the UL30 gene (Fig 5). During the acute infection time course at an MOI of 3, we found modest, yet significant, increases in 17*syn*+ viral DNA by 3 hpi (Fig 5A). These data are quantifed in Table 3. This trend continued into modeled latency (Fig 5B) where we measured a 3-fold increase in HSV-1 copy numbers compared to 1 hpi. The fold change from the 24hr acute time point to the latency time point is calculated in Table 4. In contrast, vero cells infected at a similar MOI with strain 17*syn*+ shows a 100-fold increase in viral DNA levels by 12 hpi [68] and this result has been recapitulated with other strains in other primary and immortalized epithelial and fibroblast cell lines [69]. Together, these data illustrate the relative resistance of LUHMES neurons to HSV-1 viral DNA replication compared to other cell types.

**Table 3:** The fold change values shown in Fig 7 for the acute time course.

Kinetic Class	mRNA	3hr	6hr	24hr
Immediate Early (α)	α4	3.66****	7.1****	13.8****
	α0	1.1(ns)	1.7****	7.9****
	α27	4.2**	11.5****	45.3****
	α22	3.5****	8.7****	30.3****
Early (β)	UL29	1.3(ns)	5.6***	35.8****
	UL23	1.9(ns)	8.0****	34.5****
	US3	1.2(ns)	2.1****	5.3****
Late (γ)	UL48	1.1(ns)	1.9***	7.3****
	UL44	1.2(ns)	2.2****	3.3****
	γ_1_34.5	0.9(ns)	1.0(ns)	10****
	LAT	1.2(ns)	1.6*	4.4****
	GAPDH	1.1(ns)	1.1(ns)	0.95(ns)

**Table 4:** Fold change in HSV-1 genome copy from the 24hr acute infection to the latency time point.

Kinetic Class	mRNA	Latent
Immediate Early (α)	α4	0.204****
	α0	0.092****
	α27	0.069****
	α22	0.124****
Early (β)	UL29	0.030****
	UL23	0.016****
	US3	0.010****
Late (γ)	UL48	0.149****
	UL44	0.102****
	γ_1_34.5	0.027****
	LAT	3.996****
	GAPDH	1.12(ns)

### Nascent transcription across entire HSV-1 genes is similar across all temporal classes during acute infection and remains high during latency

**Fig 6:**
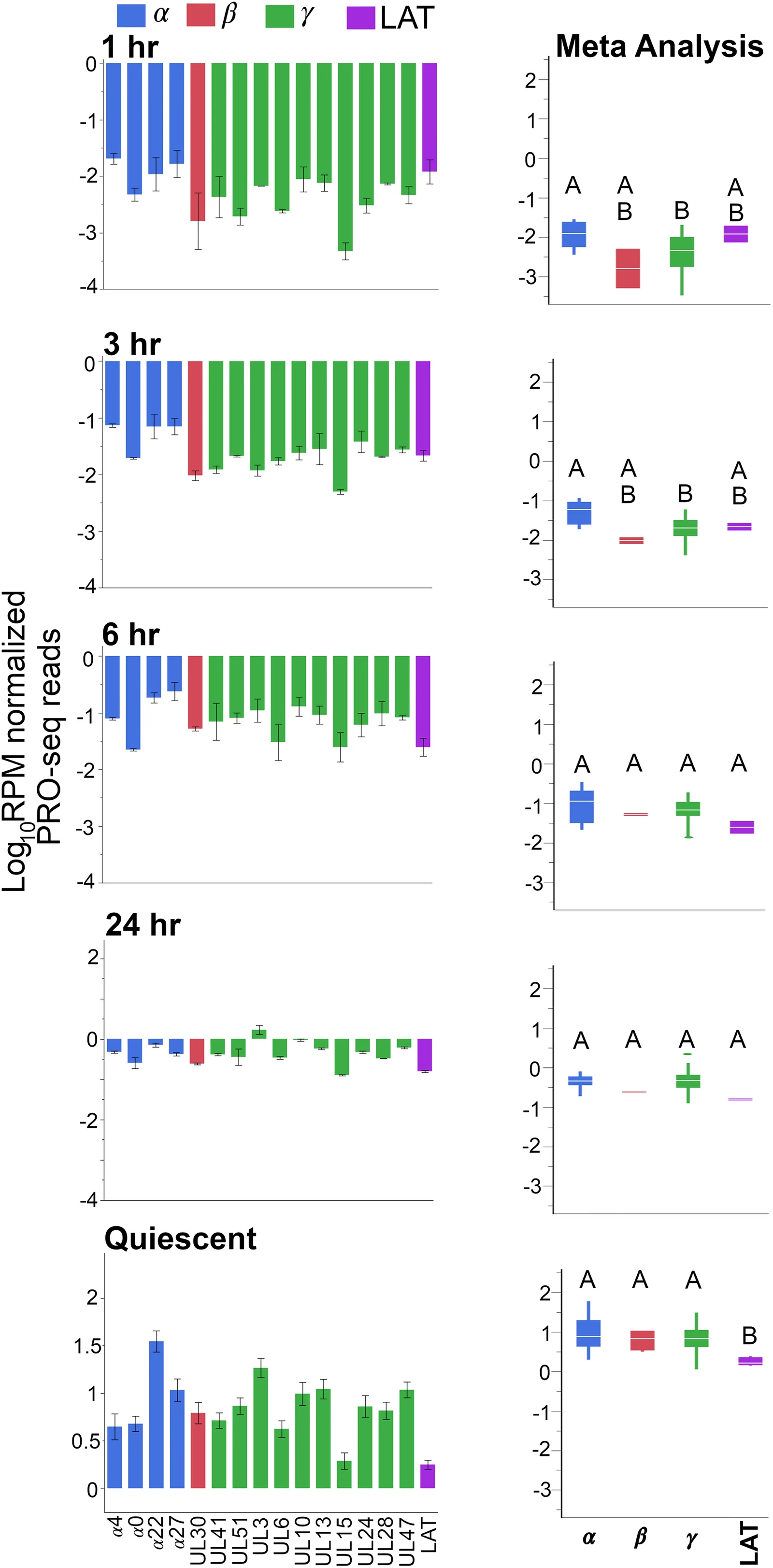
A meta-gene analysis of viral temporal gene expression in the LUHMES. Viral genes belonging to similar temporal classes were grouped, and the PRO-seq data was analyzed in a meta analysis as done previously [5]. The Log_10_ whole gene density for non-nested, viral genes with at least 1000nt distance between their poly A site and the TSS of the downstream gene shown as individual genes and grouped by temporal class for statistical purposes in a meta-analysis for all time points from the LUHMES. A Kolmogorov-Smirnov test followed by a non-parametric Wilcoxon test for each pair was used for statistics. A group was considered statistically different from another group if the p-value of the comparison was ≤ 0.05, and is indicated by the letters above each temporal class for each comparison at each time point, with groups having different letters being statistically different and groups with the same letters being statistically similar.

To assess viral transcription in LUHMES neurons, we focused on non-nested viral genes positioned 1000 nt upstream of other downstream genes in our PRO-seq analyses. Nested genes and genes situated within 1000 nt of each other were not included because the short reads from PRO-seq preclude gene assignment. For example, reads may be derived from any gene transcribed within a nested set, from a short intergenic region, from termination of an upstream gene, or initiation at the next gene downstream. The genes are color coded according to their lytic temporal class. Data are shown for each individual gene, as is a meta-analysis of each time point (Fig 6).

At 1hr post-acute infection, Pol II activity was detected on the viral genome, but at much lower densities than occurs during lytic infection of epithelial cells (Fig 6, S2 Fig) [5,45,54,64]. Beginning at 1hr and continuing through the rest of the acute time course, the viral 𝛼 genes had statistically similar amounts of Pol II activity as LAT, which is different from what occurs in HeP2 infection (Fig 6 and S2 Fig). UL30 (representing the β genes because it is the only non-nested gene of this class) had less Pol II activity than α genes at both 1 and 3 hr post infection similar to the classical cascade seen in epithelial cells [5].

Surprisingly, transcriptional activity on lytic genes remained high during latency and was more active than on the region of the genome encoding the LAT. Moreover, all lytic genes were transcribed at roughly equal levels (Fig 6). These observations suggest that in the LUHMES neurons, and likely neurons in general, the viral genome is much more transcriptionally active then previously appreciated.

### Pol II activity does not necessarily correlate with steady-state viral mRNA levels detected by RT-qPCR

**Fig 7:**
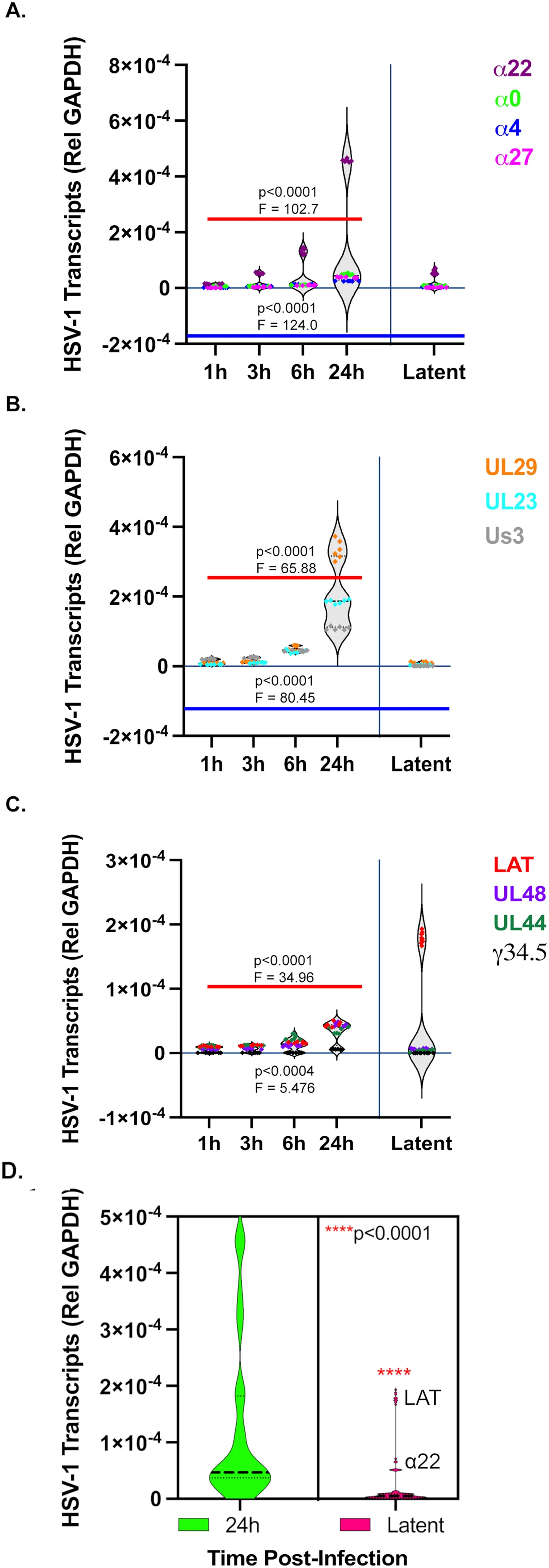
The total relative steady-state HSV-1 transcripts over time are shown. Steady-state RNA levels of select viral genes measured by RT-qPCR, normalized to human GAPDH, and grouped by kinetic class: Immediate Early **(A)**, Early **(B)**, and Late (**C**) along with the latency associated transcripts (LAT, **C**). **(A-C)** Ordinary one-way ANOVA was performed for all genes across all timepoints and the overall statistical significance is indicated on the violin graphs (**red line**: p and F values for the 1-24h acute phase; **blue line**: p and F values for entire acute-latent experiment). Statistical details along with fold change from 1h for each transcript spanning the acute infection can be found in **Table 1**. **(D)** Normalized viral transcripts from all kinetic classes, including the LATs, were compiled for the 24h acute and latent infection timepoints. Overall steady-state viral RNAs were found to be significantly lower at the latent timepoint (Welch’s t test, two-tailed; 24h sample size = 66 with mean = 0.0001208, Latent sample size = 99 with mean = 2.516E-05; difference between means Latent-24h = -9.564e-005 ± 1.815e-005). Statistical details along with fold change for each transcript spanning the 24h to latent infection can be found in **Table 3**.

To correlate transcription with steady-state RNA levels as an estimate of RNA velocity [70], we isolated total RNA from infected LUHMES neurons and determined levels using RT-qPCR (Fig 7). Of the viral α genes, α22 was the most abundant steady-state transcript at 3hr post-infection (Fig 7A), US3 was the most abundant steady-state viral β gene transcript at 24hrs (Fig 7B), and LAT(γ) was the most abundant viral γ gene transcript at 24hrs (Fig 7C). These observations were consistent with the PRO-seq detection of active transcription from these genes at 1hr (Fig 6). One caveat is that we could not exclude the possibility that these stable, RNAs were delivered to the neuron during virion entry [71–74]. The α22 gene also yielded the most steady-state transcripts of all viral genes through 24 hr (Fig 7A-7C). Consistent with the PRO-Seq data, there was a burst of steady-state transcripts at 6hpi from genes belonging to different temporal classes, including UL23 (β), UL29(β), and UL44(γ) (Fig 7B and 7C). Steady-state transcript levels increased during acute infection in LUHMES neurons (Fig 7A-7C). At 24hpi, UL29, UL23, US3, α0, α4, α27, and LAT also had detectable steady-state levels in the LUHMES neurons. Thus, the pattern of transcription and RNA accumulation in LUHMES neurons is dissimilar to the classical ordered expression of viral genes in epithelial cells.

As expected, during latency, the LAT intron was the most abundant steady-state RNA species of all viral transcripts analyzed (Fig 7D). These data verify that the latently infected LUHMES neurons express steady-state LAT to high levels consistent with observations in other model systems. The high level of steady-state LAT is likely due to its high stability as a lariat structure, and slow accumulation over time afforded in part by the continued, yet limited amount of transcription compared to other viral genes (Fig 6). In contrast, steady-state levels of viral transcripts other than LAT were lower than was observed at the 24hr acute infection (Fig 7C and 7D).

### Promoter-proximal pausing on the viral α4 and α0 genes during latency

One possible explanation for a gene with high transcriptional activity, but little detectable steady-state RNA levels, is that Pol II initiates but is unable to complete transcription of the gene due to processivity blocks within the gene body [37]. We therefore used the PRO-seq data to examine the pattern of Pol II progression along individual viral genes analyzed in the whole gene probe analysis shown in Fig 6.

**Fig 8:**
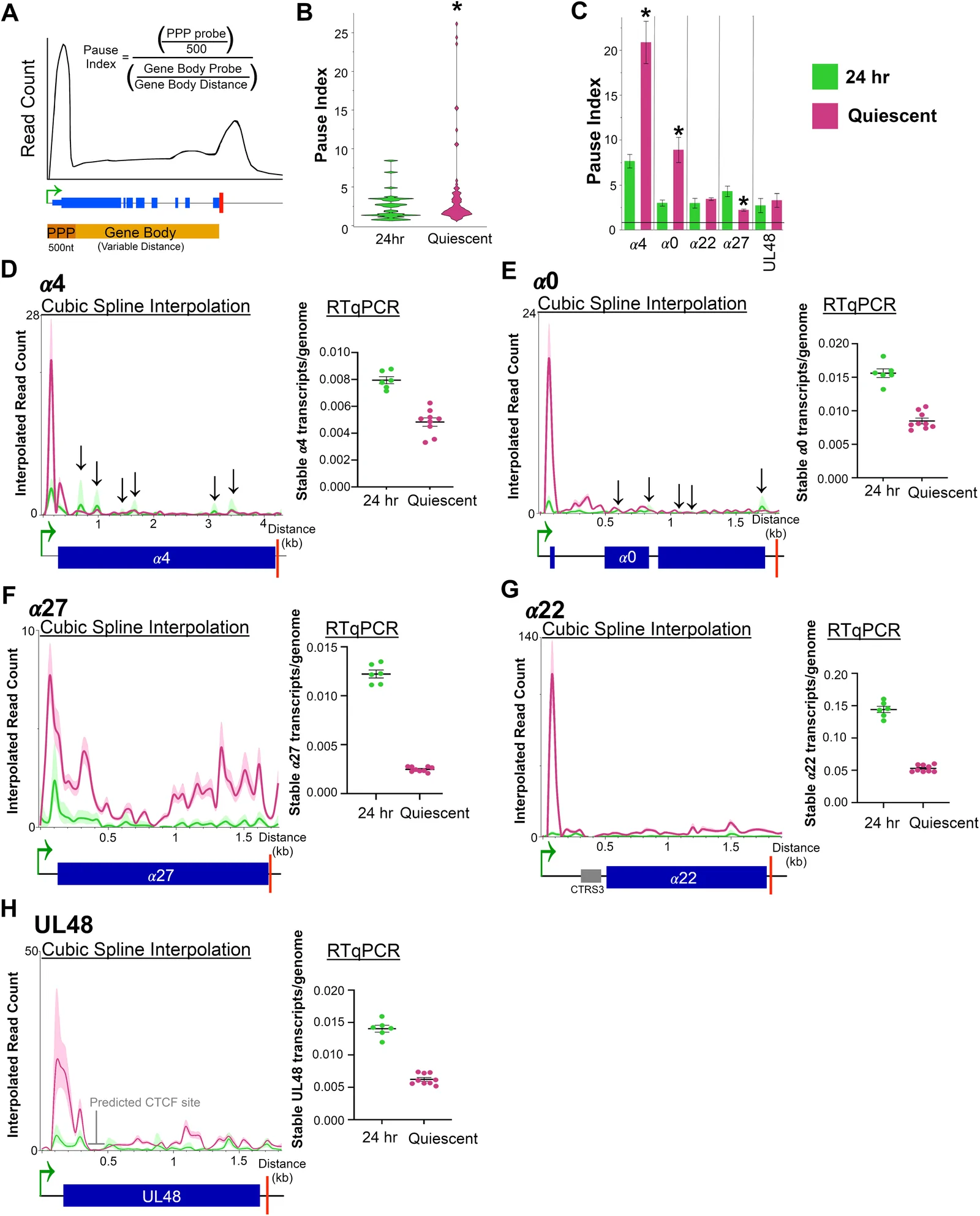
Pol II distribution along specific viral genes in the 24hr acute infection (lime) and latent (cerise) infection in LUHMES. **(A)**The pause index calculation used for the viral gene analysis. **(B)** A meta-pause index analysis of all non-nested viral genes. **(C)** Pause indices for individual viral genes of particular interest identified from the meta-analysis. A pause index of 1 (horizontal line on the y-axis) or greater indicates promoter-proximal pausing. **(D-H)** The cubic spline interpolations of the data are shown for individual viral genes along with their steady-state transcript levels as detected by RT-qPCR. The viral genes shown are α4 **(D)**, α0 **(E)**, α27 **(F)**, α22 **(G)**, and UL48 **(F)**. An ANOVA analysis was performed for the pause index calculations in B and C and a star indicates a significantly different pause index in the latent samples compared to the 24hr acute samples, with a p-value < 0.05. The cubic spline interpolations of the data for each gene are shown as the dark line, with the bootstrap confidence of fit for the spline indicated with the lighter shaded areas around the spline.

For the viral genes, we chose a promoter-proximal probe 500nt downstream of the transcription start site of each gene (Fig 8A). We chose this window because it captures the variable nt length of the promoter-proximal pause region on most viral genes based on a by-eye evaluation of the analyzed genes in IGV. This analysis was previously described for a viral mutant lacking expression of ICP27 [45].

Using a meta-analysis, we first analyzed the pause indices from the non-nested viral gene subset (Fig 8B). The distribution of the data indicated that Pol II pausing on most viral genes was similar at 24hr acute infection as it was during latency. However, predominant outliers in this analysis pushed the mean of the viral gene pause indices in the latent samples significantly higher than that for the 24hr post-infection samples (Fig 8B). Further analysis indicated that α4 and α0 were the only genes with significantly increased promoter proximal pausing during latency compared to the 24hr acute infection (Fig 8C). We also noted that promoter-proximal pausing on α27 during latency was significantly decreased compared to the 24hr acute infection (Fig 8C). No other viral genes exhibited differences in promoter-proximal pausing at 24 hpi vs latency. As examples of this group, the pause indices of α22 and UL48 are shown (Fig 8C).

We then compared the cubic spline interpolations of the PRO-seq data across each of the individual gene loci depicted in Fig 8C (Fig 8D-8H). The interpolations are shown as the dark colored line, and the shaded bootstrap confidence of fit representing 2000 iterations of the data is shown as the lighter shaded areas around the spline. The boot-strap confidence of fit interpolations incorporate variability of the PRO-seq data unlike simple IGV visualizations [45]. We also include RT-qPCR analysis of the steady-state mRNA levels next to each spline interpolation.

For both the α4 and α0 genes the cubic spline interpolations indicated higher transcription from the promoter proximal pause region in the latent samples compared to the 24hr acute samples. These two genes also had clear instances within their gene bodies where the 24hr acute interpolation was at the same level or above the latent interpolation (arrows, Fig 8D and 8E), indicating more Pol II activity in the gene bodies at the 24hr acute time point than during latency. The data was less variable in this region so the shading designating the confidence interval was not visible. This observation combined with the lower promoter-proximal pause peak height in the 24hr acute samples further confirmed significantly higher pausing on these two genes during latency than following a 24hr acute infection. Considering that much of the Pol II activity is limited to the 5’ region of α4 and α0 during latency (Fig 8C–8E), the increase in promoter-proximal pausing on these genes likely plays a role in the lower steady-state RNA levels of both α4 and α0 detected by RTqPCR (Fig 7A and RT-qPCR data shown in Fig 8D and 8E).

Interestingly, the observed blocks to elongation seen at the promoter-proximal pause region for α4 and α0 do not explain the decreased levels of steady-state a27 RNA measured at the latent time point compared to the acute infection (Fig 8C and 8F). The increased levels of initiation and decreased levels of promoter-proximal pausing on α27 in latency compared to the 24hr acute infection led to more Pol II activity at the poly-A site, suggesting that any decreases in steady-state mRNA levels of α27 likely resulted from one of the many post-transcriptional processes that control RNA velocity and steady-state mRNA levels in the cell [35]. High levels of activity of this gene could also be due to readthrough from upstream genes.

Most viral genes, like α22, exhibited increased initiation during the latent infection and the same rate of promoter-proximal pause release compared to the acute infection (Fig 8C and 8G). Activity in the gene body of α22 during latency was higher than in α0 and α4. Unlike those genes, no region of α22 contained similar levels of activity at the latent vs acute infection time point. These features correlated with more Pol II at the poly-A site in the cubic spline interpolation, yet resulted in fewer steady-state α22 RNA levels during latency compared to the acute infection. Intriguingly, α22 has a well-defined CTCF binding site shortly downstream of the promoter proximal pause site (Fig 8G), and although the gene body of α22 was transcribed during latency, the presence of CTCF at this locus likely acts as a barrier to proper α22 mRNA processing [20,75–78]. Like α27, the decrease in steady-state RNA levels of α22 is likely controlled post-transcriptionally.

For some genes, like UL48, the cubic spline interpolations showed regions of the gene body where Pol II accumulated to higher levels during latency than following the acute infection. This suggests that Pol II processivity blocks downstream of the promoter-proximal pause site could be responsible for decreased steady-state UL48 RNA during latency (Fig 8H). Notably, a sequence-predicted CTCF site within the UL48 gene body may impose a barrier to UL48 mRNA processing, a result which requires further investigation.

### The internal repeat region, the unique long and unique short regions of the genome have distinct transcriptional signatures

**Fig 9:**
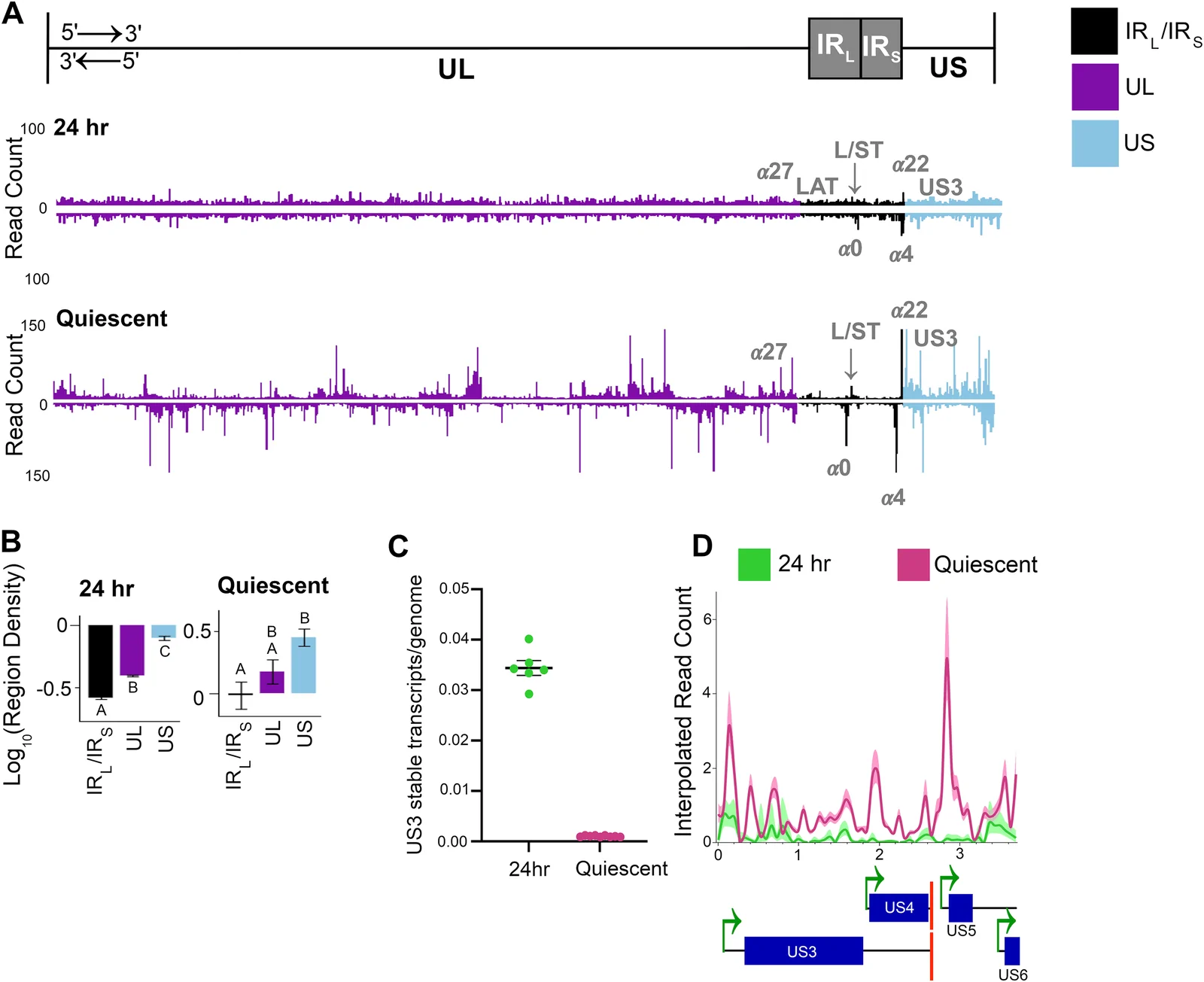
Analysis of transcription across the unique long (UL), unique short (US) and internal repeat (IR_L_/IR_S_) regions of the viral genome. **(A)** IGV images of the RPM normalized PRO-seq read distribution across the HSV-1 genome and quantification of total transcription in each viral gene region at the 24hr and acute time points. The UL, IR_L_/IR_S_, and US regions are color-coded in purple, black, and teal, respectively. **(B)** The indicated regions were probed, and Log_10_ transformed read density for each area was calculated and compared. A one-way ANOVA followed by Tukey post hoc test of all comparisons was used for the analysis. **(C)** A comparison of the US3 steady-state mRNA levels for the 24hr and latent LUHMES infections. **(D)** The cubic spline interpolations of the 24-hour and latent infections for the US3 to US5/6 gene region. The cubic spline interpolations of the data are shown as the dark lines, with the bootstrap confidence of fit for the spline indicated with the lighter shaded areas around the spline.

We noted that both viral genes with increased pausing and increased initiation during latency were located within the internal repeat region (black region Fig 9A), which is flanked by and contains multiple CTCF binding sites (Fig 9A) [51,75–78]. To investigate this observation further, we compared the transcriptional activity on entire genomic components on both strands of the viral genome. As was observed with single gene probes, the Pol II density in each region was higher in latency than in the 24hr acute infection (Fig 9B). Notably, the pattern of viral nascent RNA production suggested that transcription was much more restricted in the IR_L_/IR_S_ region than the UL or US regions at both 24hr acute infection and during latency.

### The unique short (US) component exhibits the highest transcription at both the 24hr acute and latent time points

Most genes in the US region were excluded from the single-gene analysis because they lie within nested sets that share poly A sites (Fig 6). However, the US area of the genome was of interest because it exhibited high levels of nascent RNA production at 24hr acute infection and during latency compared to other regions (Figs 9A and 9B).

To further investigate the transcriptional activity within the US region, we compared the US3 RT-qPCR data to the cubic spline interpolation of the PRO-seq data (Fig 9C and 9D). Like most steady-state viral RNAs, we found that US3 steady-state RNA levels were lower during latency compared to the 24hr acute infection (Fig 9C), yet transcription initiation and nascent RNA production was higher in latency than in 24hr acute infection (Fig 9D). During the acute infection, the cubic spline interpolation of the data showed that US3 exhibited more promoter-proximal pausing than the coterminal US4 and US5/6 in the downstream nested gene set. During the latent infection, transcription of this region contained clear promoter-proximal pausing on US4, US5, and US6 with more areas of highly active transcription downstream (Fig 9D).

Despite the significant increase in transcription initiation, the uneven distribution of Pol II activity on US3-US6 during latency suggests additional barriers to Pol II processivity/ RNA processing than at the 24-hour acute infection in LUHMES. Blocks to processivity or mRNA processing on any of the genes within the nested set could confer a decrease in steady-state US3 mRNA levels (Fig 9C and 9D).

### Transcription patterns in the IR_L_/IR_S_ region of the viral genome is unique to neurons

The IRL/IRS was the only component containing single genes with increased promoter-proximal pausing from 24hr to latent infection that correlated with decreases in steady-state transcript levels (Fig 9A and 9B). This region also encodes for neuron-specific transcripts that have been investigated significantly in mouse and rabbit animal models, including LAT, L/ST, and the neurotropic factor γ_1_34.5. Nascent RNA production from L/ST is detected only under certain conditions: early in HeP-2 infections (1.5hr and before), during global protein production inhibition with cycloheximide, or during infection with viruses lacking ICP4 [5,13,79,80]. L/ST transcriptional initiation is quickly extinguished in epithelial cell infections, likely through ICP4 binding to its promoter and interaction with other viral α proteins. This results in transcriptional silence at the L/ST transcription start site by 3 hours post-infection in HeP-2 cells [5,13].

**Fig 10:**
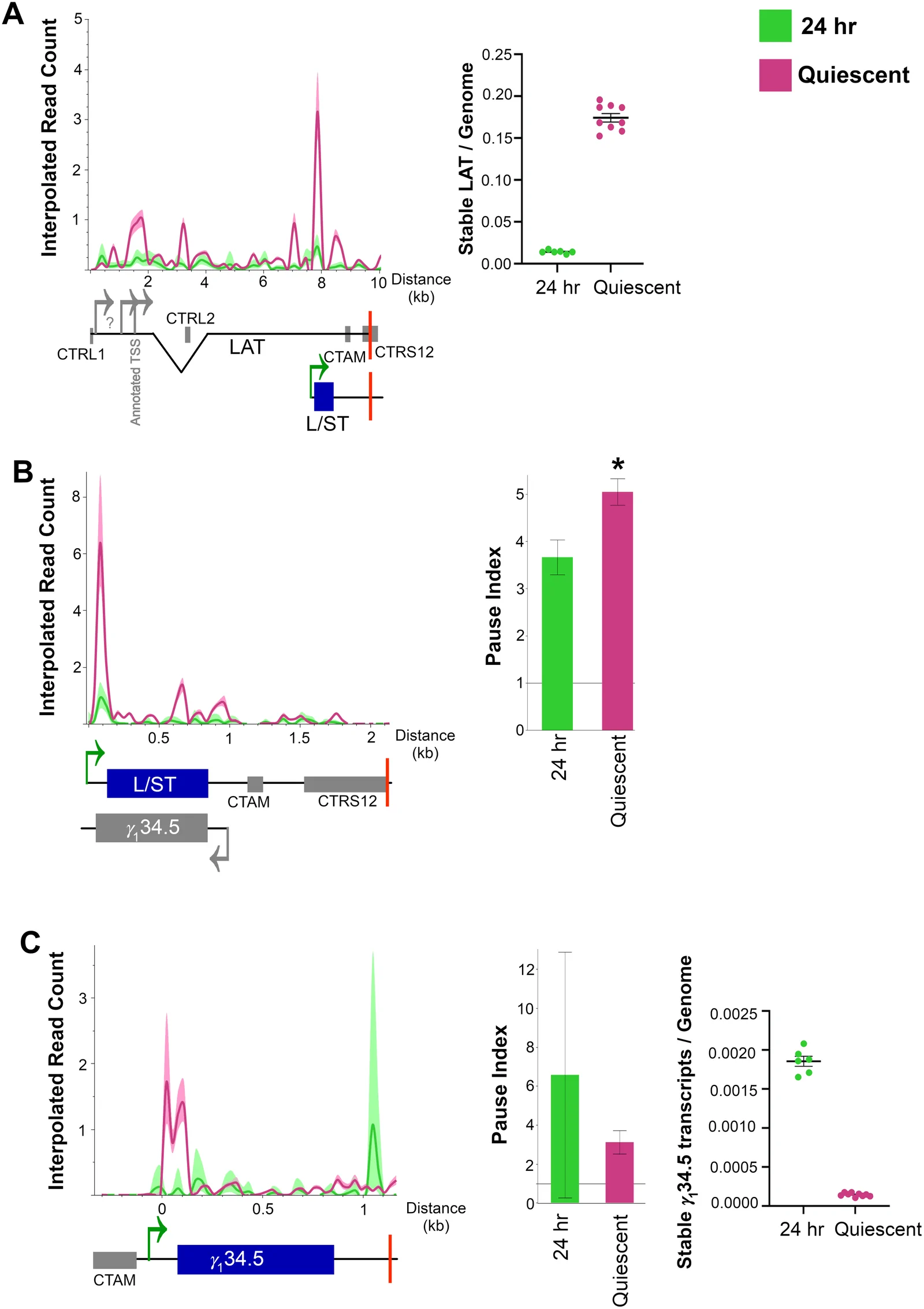
Cubic spline interpolations and pause index calculations for important viral genes associated with neuronal infection and nested within the LAT region. **(A)** A cubic spline interpolation for the entire LAT coding strand is shown, beginning at the CTRL1 CTCF binding site and ending at the CTRS12 CTCF binding site. The steady-state transcript levels of the LAT intron, measured by RT-qPCR are also shown. **(B)** The nested L/ST transcript is depicted along with the cubic spline interpolation of the data across this locus. A hypothetical pause index calculation, assuming all reads in the L/ST gene body are initiated at the L/ST transcription start site is also shown. **(C)** The cubic spline interpolation, pause index calculations, and steady-state transcript levels of the γ_1_34.5 gene. The γ_1_34.5 transcription start-site was inferred from the location of the promoter proximal pause site. The γ_1_34.5 gene is a non-nested gene lying between α4 and α0 and is antisense to the LAT and L/ST transcripts. Statistical analysis was performed using a one-way ANOVA. A star represents a p-value <0.05 between the 24hr and quiescent samples.

To investigate L/ST transcription in LUHMES, we generated cubic spline interpolations at 24hr acute infection and in latency (Fig 10). The L/ST transcript is nested within the 3’ region of the LAT locus (Figs 10A and 10B) and lies between the CTRL1 and CTRS1/2 CTCF binding sites. The γ_1_34.5 gene is encoded on the opposite strand of L/ST (Figs 10B and 10C).

During 24hr acute infection, the L/ST promoter-proximal pause peak was visible within the 3’ region of the LAT locus. However, the level of L/ST pausing was much more clearly defined during latency (Fig 10A). Final steady-state LAT levels increased significantly from 24hr acute infection to latency, and this was the only gene in which the increase in nascent RNA production from the 24hr acute infection to latency correlated with an increase in steady-state RNA. The pause index calculation for the LAT was compromised by different locations of high Pol II activity near the documented LAT transcription start site, suggesting variability in the transcription start site of this large, spliced transcript.

We then focused on the L/ST transcript in the cubic spline interpolation, assuming it was a non-nested transcript and all nascent RNA activity after the L/ST TSS initiated at this TSS (Fig 10B). With these assumptions, L/ST promoter-proximal pausing increased from the 24hr acute to the latent infection. The fact that the L/ST transcript had increased initiation and promoter-proximal pausing during latency compared to the 24hr acute infection is reminiscent of these parameters for α4 and α0. The absence of any transcription from L/ST during productive infection in epithelial cells indicates that this activity is a signature specific to the LUHMES neuronal infection, as opposed to epithelial cells.

### Promoter-proximal pausing on the γ_1_34.5 locus is only observed during latency

The γ_1_34.5 gene was also regulated differently in latency than at the 24hr acute infection. This gene does not have an annotated transcription start site. However, the transcription start site can be inferred by the appearance of the promoter-proximal pause-peak in both epithelial cell infections at 3 and 6hrs [5] and in latency (Fig 10C). The cubic spline and pause index calculations for this gene suggest that the transcription start site of γ_1_34.5 is not utilized extensively at 24 hr acute infection as the confidence intervals for the cubic spline interpolations are very wide, and the pause index calculations show large variability within replicates (Fig 10C).

These observations are dissimilar to most other viral genes presented in this manuscript, except for the LAT, which also showed a lack of consensus concerning its transcription start site at 24hr acute infection (Fig 10A). During latency, however, γ_1_34.5 showed a clear area of activity downstream from the inferred transcription start site (Fig 10C). This differed from the LAT and correlated with lower steady-state γ_1_34.5 transcripts during latency, akin to what occurred with α0 and α4. These data demonstrate possible promoter-proximal pausing on L/ST, and γ_1_34.5, accompanying the documented α0 and α4 pausing, and comprising a transcriptional signature of latency in LUHMES neurons. Collectively, these data indicate unique regulation of the genes within the repeat regions.

## Discussion

The current study analyzes transcription in a human post-mitotic HSV-1 neuronal infection/latency model. The precise mapping of active transcription (nascent transcriptomics) was not possible until the development of techniques such as PRO-seq that report the position of active RNA polymerase at nucleotide resolution. This level of detail provides information critical to understanding how latency is established and maintained on a molecular level. A caveat is that Pro-seq and ancillary sequencing techniques require large numbers of cells and report nascent transcriptional activity on a population level. Thus, the data do not discern any putative variation among cells, some of which have been observed upon both establishment and reactivation in this system [22,47,48].

Analysis of cellular transcription suggests a very different cellular environment encountered by HSV-1 upon infecting LUHMES versus epithelial cells. As expected, the uninfected LUHMES, which had been differentiated and were post-mitotic, transcribed genes related to dopaminergic signaling and cell adhesion but did not transcribe genes involved in progression of the cell cycle, nor innate immunity, among others. More surprising was that upon infection, we observed only modest Pol II recruitment to viral genomes compared to epithelial cells, and an opposite effect on promoter-proximal pausing: increased pausing on many LUHMES genes compared to the accelerated release from promoter-proximal pausing shown in previous studies on many epithelial cell genes [11,46,53]. Both traits likely relate to the relatively low expression of viral gene products in LUHMES, some of which would be expected to alter cellular transcription to favor viral gene expression.

Interestingly, LUHMES were much less permissive to viral DNA replication and transcription during acute infections, especially when compared to epithelial cells, fibroblasts, and neuroblastoma cell lines [69,81,82]. This suggests that the default pathway in mitotic cells involves the rapid production of infectious particles with ensuing cell death; infection of LUHMES, by contrast, tends towards nonproductive infection and long-term cellular survival [5,6,83,84].

Unexpectedly, we found that latently infected LUHMES exhibited more transcription, i.e., per viral genome, than was apparent during acute infection of these cells (e.g., 24 hpi). However, this increase in transcription is accompanied by decreased levels of steady-state RNA from the same genes. The LAT intron was an exception to this general observation and increased in steady-state levls over time despite increasing only modestly in transcription or nascent RNA levels, likely due to the stability of its lariat structure [85]. The discordance between increased transcription and decreased steady-state RNA suggests that regulatory steps after transcriptional initiation preclude the production of steady-state, full-length mRNA from most viral genes in LUHMES neurons. At least for α0 and α4, this mechanism likely includes promoter-proximal pausing. For other genes such as UL48, encoding VP16, poor processivity of Pol II through the gene body likely plays a role. These studies also cannot rule out roles for decreased RNA stability or poor RNA processing in LUHMES. The state of viral chromatin, CTCF binding, and genome architecture undoubtedly play important roles. For example, it has been known for some time that nonpermissive histone marks are associated with viral genomes in latently infected mouse and rabbit ganglia [15,51,86–88]. Likely, viral chromatin is locally remodeled and enriched for euchromatin to allow nascent RNA initiation [89,90].

Collectively, these results indicate that in LUHMES neurons, latency is partly maintained through mechanisms that impede the elongation or processing of previously initiated transcripts from viral lytic genes. Controlling ICP4 and ICP0 production may be particularly critical as these proteins enhance the production of most other viral genes, at least in epithelial cells [13,45,53,54]. The fact that α22 is particularly active during latency is also intriguing because its encoded protein, ICP22, has been shown to impede Pol II elongation at immediate early times [45,53,54]. Blocks to elongation are the most common regulatory mechanism to control the expression of cellular genes undergoing constant transcription in post-mitotic cells [30,91]. Inhibition of elongation has also been shown to suppress both immediate early gene expression and reactivation from latency in a mouse model [92]. It follows that viral reactivation could result from enhancing Pol II elongation on α4 and α0, allowing ICP4 and ICP0 production, and thus initiating nascent transcription of viral genes important for viral DNA replication and infectious virus production. The LUHMES neuronal culture model along with modern deep-sequencing techniques, make these hypotheses experimentally testable.

## Materials and methods

### LUHMES cell culture

LUHMES cells were purchased from ATCC (catalog no. CRL-2927) and cultured as previously described [52]. Briefly, T75 flasks (Nunclon Delta Surface, ThermoFisher Sci., catalog no. 156499) were coated with poly-l-ornithine hydrobromide (Sigma, catalog no. P3655) followed by fibronectin (Sigma, catalog no. F2006), rinsed with water, and allowed to fully air dry before cell plating. LUHMES cells were maintained in proliferation media (DMEM: F12, ATCC, catalog no. 30–2006) containing 1% N2 supplement (ThermoFisher Sci., catalog no. A1370701), 1X Penicillin-Streptomycin-Glutamine solution (ThermoFisher Sci., catalog no. 10378016) and recombinant human FGF-basic (Fibroblast Growth Factor, Peprotech, catalog no. 100-18B); reconstituted at 100μg/mL in 10mM Tris pH 7.6 plus 0.1% BSA) at 40ng/mL final concentration. To induce cellular differentiation, LUHMES were cultured in DMEM: F12 plus N2 supplement media containing 1 μg/mL tetracycline hydrochloride (Sigma catalog no. T7660), 1mM N6,2′-O-Dibutyryladenosine 3′,5′-cyclic monophosphate sodium salt (Sigma, catalog no. D0627) and 2ng/mL recombinant human GDNF (Glial cell-derived neurotrophic factor; R&D Systems, catalog no. 212-GD-010). All experiments were conducted with LUHMES neuronal cells after 8 days post-induction of differentiation.

### Infections

LUHMES were plated at 40,000 cells/cm2 onto coated dishes and flasks, and cells were allowed to proliferate for two days, at which time they were switched into differentiation media. At 7 days post-differentiation (9 days post-plating), For the latency experiments, LUHMES neuronal cultures were pre-treated overnight with 50µM Acyclovir (ACV). For the acute phase infections, ACV was not included at any timepoint. Both the acute and latent infections were conducted using HSV-1 strain 17*syn+* at an MOI of 3 on day 10 post-plating (8 days post differentiation). Latency was established by including ACV in the media for the first 2 days of infection after which the media was changed and the LUHMES were cultured in the absence of ACV for the remainder of the experiment. Nuclei were prepared from acutely infected LUHMES neuronal cultures at 1, 3, 6, and 24hr post-infection, and the latently infected cultures collected at 10 days post-infection (8 days post-ACV removal). There were five independent PRO-seq experiments conducted for the latently infected cultures and two independent PRO-seq experiments conducted for each acute infection time point. Each independent experiment consisted of five T75 flasks with an approximate number of neurons per experiment totaling 3-3.5x10^8^.

### Nuclei Isolation

Nuclei were isolated at the indicated times post-infection, as described previously [43,46,93]. PRO-seq was performed following previously published protocols for HSV-1-infected HeP-2 cells [5,46,53]. Frozen nuclei (100 µl) were brought to 37°C. *Drosophila* S2 nuclei were spiked into the infected LUHMES nuclei at a ratio of 1/1000 *Drosophila*/Human nuclei. Pre-warmed run-on reaction buffer (10 mM Tris-HCl [pH8], 300 mM KCl, 1% Sarkosyl, 5 mM MgCl_2_, 1mM DTT, 100 μM biotin-11-ATP, -CTP, -GTP, and -UTP [Perkin-Elmer], and 0.4 U / μL of RNAse Inhibitor) was added to each sample. Reactions were incubated at 37°C with constant shaking for 3 minutes. Reactions were stopped by adding 1.8 mL of Trizol LS (Thermo Fisher). RNA was isolated by adding 520 μL chloroform to each resuspended sample. Incubation times and centrifugation times were performed following the manufacturer’s guidelines. RNA was precipitated with a 2:1 volume of ethanol, and the pellets were washed and stored in 700 μL of 75% ethanol) before sequencing library preparation.a

### PRO-seq library preparation

The biotinylated RNA was resuspended in nuclease free water and hydrolyzed with 0.2N NaOH in a final volume of 30 μL. After 20 min, the NaOH was neutralized by adding 1M Tris-HCl (pH 6.8), and samples were run through a Bio-Rad P-30 column to remove unincorporated biotinylated nucleotides. Following previously published protocols, biotinylated RNA was isolated using streptavidin M280 Dynabeads (Thermo Fisher) [5,46,53].

The 3’RNA adapter (5’Phos-GAUCGUCGGACUGUAGAACUCUGAAC-3’InvdT) was ligated to the RNA using 10 U/μL of T4 RNA ligase I (New England BioLabs [NEB]) as described [46]. After an additional round of M280-Streptavidin bead binding and washing, RNA 5’-pyrophosphohydorlase (RPPH) (NEB) was used to remove any 5’ caps on the biotinylated RNA [46]. After cap removal, T4 polynucleotide kinase (PNK) (NEB) in its associated reaction buffer [46] was added directly to the RPPH mix to repair the 5’-hydroxyl group. Trizol purified RNA was ligated to the 5’adapter (5’-CCUUGGCACCCGAGAAUUCCA-3’). RNA was reverse transcribed and PCR-amplified using barcoded sequencing primers (Table 5) for 13 cycles, which was experimentally determined, as previously described [5,46]. Libraries were run through an 8% polyacrylamide gel, and fragments of 140nt and larger were extracted from the gel and submitted for sequencing [46].

**Table 5:** Primers used for barcoded PRO-seq library preparation. Libraries were amplified using RP1 and one of the RPI barcoded primers.

Index Primer	Sequence
RP1	5’-AATGATACGGCGACCACCGAGATCTACACGTTCAGAGTTCTACAGTCCGA-3’
RPI1	5’-CAAGCAGAAGACGGCATACGAGATCGTGATGTGACTGGAGTTCCTTGGCACCCGAGAATTCCA-3’
RPI2	5’- CAAGCAGAAGACGGCATACGAGATACATCGGTGACTGGAGTTCCTTGGCACCCGAGAATTCCA-3’
RPI3	5’-CAAGCAGAAGACGGCATACGAGATGCCTAAGTGACTGGAGTTCCTTGGCACCCGAGAATTCCA-3’
RPI4	5’- CAAGCAGAAGACGGCATACGAGATTGGTCAGTGACTGGAGTTCCTTGGCACCCGAGAATTCCA-3’

### Sequencing

The concentration of the prepared libraries was determined using the qubit fluorometric quantification system (Thermo Fisher). The barcoded libraries from the quiescent infections were mixed according to Illumina guidelines and single-end sequenced on the NextSeq500 platform (Illumina) as previously described [53]. The barcoded libraries from the acute infections were pair-end sequenced on the NovaSeq X Plus PE150 platform. For comparison to the latency infections, the paired-end data from the acute infections was analyzed as single-end data. Sequences were aligned to a concatenated index file containing the hg38 human genome, the dm3 *Drosophila melanogaster* genome, and the HSV-1 strain 17 genome (NC_001806.2) with deleted terminal repeats (https://github.com/Baines-Lab/Public/tree/main/HSV-1) using the Danko PRO-seq2.0 pipeline (https://github.com/Danko-Lab/proseq2.0) [94]. To provide nucleotide resolution, this algorithm uses cut adapt to trim the adapter sequences and bwa to perform the alignment, then converts the BAM file into bigwig (*.bw) files that report the read counts only for the 3’ most nucleotide in the read [43,44].

### PRO-seq data normalization

All PRO-seq data was probed and quantified using SeqMonk Software [95,96] and the  .bam files from the PRO-seq 2.0 alignment. The HeP-2 data sets were normalized with cellular RPM and *Drosophila* spike-in values. We and others previously demonstrated that cellular reads fluctuate as HSV-1 infection progresses in epithelial and fibroblast cells [46,97]. Viral read data from the LUHMES infections were normalized to the cellular RPM amounts, as these values were more consistent over all experimental time points (Table 1). They also showed more consistency than the *Drosophila* spike-in, likely because we reduced the amount of spike-in used for the LUHMES infections to increase the sequencing depth of viral reads (Table 1). For the cellular RPM normalization, the total human PRO-seq reads for each sample were divided by 1,000,000 and then used to determine the scaling factor for the data. The data from each sample was scaled to match the sample with the lowest cellular reads before being divided by the probe length for density calculations.

### Cellular gene DESeq2 analysis

The SeqMonk Software [95,96] and its integrated Ensembl gene annotation database were used to probe the  .bam files for human reads aligning between the transcription start site (TSS) and transcription termination signal (TTS) of genes longer than 16,000 nt, which ensured an adequately long gene body to accommodate the expanded 2,000 nt promoter-proximal pause window. Genes were included if they had more than 200 reads per gene, with a read density of at least 0.01 in their bodies. The DESeq2 function [98] in the SeqMonk Software was used to identify genes with significant differences between nascent RNA production in the two cell types. For this analysis, a gene with a Log_2_ Fold change ≥ |1| and a p-value ≤ to 0.05 was considered significantly different in LUHMES compared to HeP-2 cells.

### Gene ontology analysis

The cellular genes with either an HSV-1 induced increase or decrease in transcriptional activity were grouped and then used as input for the gene ontology analysis for biological function using the DAVID Bioinformatics tool (https://davidbioinformatics.nih.gov/) and a functional annotation analysis using the Functional Annotation tool and the UP_KW_Biological Process and GOTERM_BP_Direct databases [99]. Groups of genes were considered enriched in the data set if their respective enrichment score was above 0.5 and if they had a p-value ≤ 0.05.

### Visualization of cellular gene PRO-seq Data using integrative genomics viewer (IGV)

The RPM normalized  .bw files from the PRO-Seq2.0 output for each replicate were overlaid with other replicates from like time points using the bar chart function in IGV [100]. The settings were adjusted to view the maximum value for each bin and the y-axis scale was adjusted for each individual time point to best observe the locations of the peak heights across *DDX3X*.

### Pause index and meta-analysis

The pause indices used in this manuscript are similar to previously descibed pause indices [53]. Because HSV-1 infection increases the window of promoter-proximal pausing, we extended the probe for the numerator (PPP probe) of the pause index to include the first 2000nt of the cellular gene, starting at the TSS site. For the viral genes we used a PPP probe of 500nt [45]. The gene body probe comprised a variable distance to the poly-A signal. Once probed, the normalized read counts for each area were divided by the probe’s length to indicate the read density. The PPP read density was then divided by the gene body density to yield the pause index. A pause index greater than 1.0 indicates a higher read density in the PPP region of the gene than in the gene body, indicating pausing. The effect of HSV-1 infection on pausing was assessed for each sample, and statistical differences after each time point and in each cell type were made using a Mann-Whitney U-test. A p-value ≤ 0.05 was considered significant. Cellular genes were included in the analysis if they met the criteria for the DESeq2 analysis, without the limitation of being found in the HeP2 data set.

### Viral genome copy number analysis

Total cellular nucleic acid was extracted from HSV-1 17*syn*+ infected LUHMES using the Quick-RNA Miniprep Kit (Zymo Research, catalog no. R1055), followed by isolation of DNA from the genomic DNA spin columns included in the kit with buffers from the Quick-DNA Miniprep Kit (Zymo Research, catalog no. D3024).

To analyze HSV-1 genome copies, ABI Taqman assays were conducted on a Roche LightCycler 480 using DNA concentration was measured using a NanoDrop spectrophotometer. 20ng of DNA was PCR-amplified in a final volume of 10uL containing Applied Biosystems TaqMan Master Mix along with ABI-designed primers and probes spanning the UL30 gene: forward 5’- AGAGGGACATCCAGGACTTTGT-3’, reverse 5’- CAGGCGCTTGTTGGTGTAC-3’, probe 5’- ACCGCCGAACTGAGCA-3’. Relative HSV-1 viral genome copies were calculated based on the standard curve method. Purified DNA from HSV-1 infected rabbit skin cells was used to create a standard curve of serial dilutions covering the cycle threshold sample range and qPCR efficiency was calculated for UL30 (E = -1 + 10^(-1/slope)^). Standard curves were included for each PCR run and viral genome quantification was performed from standards showing PCR efficiencies between 95–105%.

### Viral gene PRO-seq data analysis

#### Whole gene density analysis.

Genes were probed from their annotated transcription start sites to their curated polyadenylation signals and given temporal class designation based on an extensive literature review conducted previously [5]. The RPM normalized read counts for each probe were divided by the probe length and then plotted for each gene or temporal class for the meta-analysis. For the single gene analysis, statistics were performed on the Log_10_ transformed data, comparing read density between genes from each time point using a one-way ANOVA followed by a Tukey posthoc analysis with the SAS JMP software [101]. Only non-nested genes separated by at least 1000nt from their downstream, neighboring gene which have a temporal class designation were analyzed and grouped. The average whole gene density from the group of genes was then compared using a Kolmogorov-Smirnov test to account for the large variations in the number of genes included in each category. A p-value < 0.05 was considered significant for the ANOVA and Kolmogorov-Smirnov tests. Categories with different characters are statistically different (designated A, B, etc), while categories with the same characters are not statistically different.

#### Pause index calculations.

The read counts from the PPP and body regions were obtained with probes for the 500nt region downstream of the transcription start site for the PPP probe and the remaining variable gene distance to the transcription termination signal for the gene body probe on viral genes [45]. The data was then divided by the respective distance of each probe to get the read density. The read density for the PPP region was then divided by the read density for the gene body region to get the pause index. A Mann-Whitney U test was used for statistical analysis with a p-value < 0.05 considered significant.

#### Cubic spline interpolations.

The pyBigWig tool from deep tools was used to extract read count data for each nucleotide for every given window, specifically tailored to each individual gene using the  .bw- RPM normalized aligned files from the PRO-seq 2.0 output, and used to generate cubic spline interpolations of the Pol II distribution along the given locus [45,53,54]. This approach overcomes the limitations of viewing the nt resolution PRO-seq data with a bin function in IGV. Interpolations were created from combined nt read counts from each biological replicate in the indicated experimental set [45]. The interpolations were plotted using the spline function in the SAS JMP pro software for Macintosh x64 (JMP, version 16.0.0; SAS Institute Inc., Cary, NC). The λ for the spline for each window was set to a window-specific value where $\lambda =\frac{\text{2}}{windowlength(nt)}$. The shaded area around the spline is the boot-strap-derived confidence of fit of the interpolations representing 3000 different iterations of the data [45].

#### Integrative genomics viewer (IGV) representations of the data.

The PRO-seq2.0 aligned RPM  .bw files were used for the IGV presentation of the data. With like replicates overlaid in the viewer. Y-scales were chosen to best represent the overall genome density and individual gene peak heights. Color divisions of the genomic regions were added after IGV produced the image.

### Reverse transcription qPCR

RNA samples were treated with TURBO DNase for 30 min at 37°C followed by incubation with DNase Inactivation Reagent for 5 min at room temperature (Thermo Fisher Sci., TURBO DNA-*free* Kit, catalog no. AM1907). Reverse transcription was performed on 1ug total RNA in 25mL reactions using the High-Capacity cDNA Reverse Transcription Kit (Applied Biosystems, catalog no. 4368814) with random hexamers per the manufacturer’s protocol. The cDNA was diluted 1:50 and PCR was performed with 4.5uL of diluted cDNA using Applied Biosystems TaqMan assays using the following RT-qPCR primers and probes sequences: LAT intron forward 5’-CGCCCCAGAGGCTAAGG-3’, reverse 5’-GGGCTGGTGTGCTGTAACA-3’, probe 5’-CCACGCCACTCGCG-3’; ICP4 forward 5’-CACGGGCCGCTTCAC-3’, reverse 5’-GCGATAGCGCGCGTAGA-3’, probe 5’-CCGACGCGACCTCC-3’; ICP0 cDNA forward 5’-CACCACGGACGAGGATGAC-3’, reverse 5’-GGCGGGCGGTACGT-3’, probe 5’-ACCTGGACGAAGCAGACT; ICP27 forward 5’ GCCCGTCTCGTCCAGAAG 3’, reverse 5’-GCGCTGGTTGAGGATCGTT-3’, probe 5’-CAGCACCCAGACGCC-3’; ICP22 forward 5’- CCAGCCCTGGCTGTCT-3’, reverse 5’GCCCGGGCGTTCCA-3’, probe 5’- TCGTGGCCGATACCAG-3’; Tk forward 5’-CACGCTACTGCGGGTTTATATAGAC-3’, reverse 5’-GGCTCGGGTACGTAGACGATAT-3’, probe 5’-CACCACGCAACTGC-3’; ICP8 forward 5’-CACCAGGTTGCGCATCAG-3’, reverse 5’-CTGCATACGGTGGTGAACAAC-3’, probe 5’-ACCTCGCGGTCCACG-3’; Us3 forward 5’-GTATACCACGACCGTCGACAT-3’, reverse 5’-ACGGCAGTCTCGAAGATCAC-3’, probe 5’-CAGACCGGCGCTCCAA-3’; VP16 forward 5’- CTTGGTCGACGAGCTGTTTG-3’, reverse 5’- GCCCCGTTGCGTACAG-3’, probe 5’- CCGTCCGCGTTCATG-3’; gC forward 5’- CCTCCACGCCCAAAAGC-3’, reverse 5’- GGTGGTGTTGTTCTTGGGTTTG-3’, probe 5’- CCCCACGTCCACCCC-3’; ICP34.5 forward 5’- GGGCTGACCCCTCCCA-3’, reverse 5’- TGCTCCGCGGTGACG-3’, probe 5’- CCCCTCGCGCCCCT-3’. Relative HSV-1 viral transcript copies were calculated based on the standard curve method as decribed in the Viral Genome Copy Number Analysis section. 2ug of total RNA was reversed transcribed and cDNA was prepared for standards using serial dilutions covering the cycle threshold sample range. qPCR efficiency was calculated for each primer/probe set as described for quantification of viral genome copy number. Only standard curves with efficiencies between 95–105% were used for the analysis.

### Previously published PRO-seq data sets used in this analysis

This manuscript uses previously published PRO-seq data sets for HeP-2 cells infected with HSV-1(F). These data sets can be accessed with the following Gene Expression Omnibus (GEO) accession numbers: 1) GSE202363 for the 1.5hr samples GSM6112026, GSM6112027, and GSM6112028 and 2) GSE130342 for the 3hr samples GSM3736429, GSM3736430, and GSM3736431 and 6hr samples GSM3736432, GSM3736433, and GSM3736434. These samples were chosen because they all had Drosophila spike-ins [5,54].

## Supporting information

S1 Table Genes analyzed in the meta-analysis of promoter-proximal pausing consequential to HSV infection. Criteria for inclusion included actively transcribed genes in mock infected human HEp-2 epithelial cells and LUHMES neurons with canonical eukaryotic transcription profiles. These profiles included promoter proximal pausing, no known overlapping genes, and no active genes within 20 Kb downstream of the transcription termination site.
(XLSX)

S1 Fig A re-representation of previously published PRO-seq data using HeP-2 cells and the HSV-1 F strain, as a demonstration of how different DDX3X transcription and promoter proximal pausing are altered by infection in a different cell type. A detailed description of the data sets used in the re-analysis can be found in the materials and methods section [46,54]. (**A**) Drosophila spike-in normalized PRO-seq reads aligning to the DDX3X locus are shown for the Mock, 1.5, 3, and 6 hr time points. (**B**) A pause index analysis of the DDX3X locus over the infection time course. (**C**) A meta-analysis of how HSV-1 infection alters promote-proximal pausing on 579 cellular genes that are expressed in both LUHMES and HeP-2 cells.
(TIF)

S2 FigThe Log_10_ whole gene density for non-nested, viral genes with at least 1000nt distance between their poly A site and the TSS of the downstream gene shown as individual genes, and grouped by temporal class for statistical purposes in a meta-analysis for all time points in HeP-2 cells infected with HSV-1 F strain at an MOI of 5.A Kolmogorov-Smirnov test followed by a non-parametric Wilcoxon test for each pair was used for statistics. A group was considered statistically different from another group if the p-value of the comparison was ≤ 0.05, and is indicated by the letters above each temporal class for each comparison at each time point, with groups having different letters being statistically different and groups with the same letters being statistically similar. A detailed description of the data sets used in the re-representation can be found in the materials and methods [46,54].(TIF)
